# G = MAT: Linking Transcription Factor Expression and DNA Binding Data

**DOI:** 10.1371/journal.pone.0014559

**Published:** 2011-01-31

**Authors:** Konstantin Tretyakov, Sven Laur, Jaak Vilo

**Affiliations:** 1 Institute of Computer Science, University of Tartu, Tartu, Estonia; 2 Quretec, Tartu, Estonia; Dana-Farber Cancer Institute, United States of America

## Abstract

Transcription factors are proteins that bind to motifs on the DNA and thus affect gene expression regulation. The qualitative description of the corresponding processes is therefore important for a better understanding of essential biological mechanisms. However, wet lab experiments targeted at the discovery of the regulatory interplay between transcription factors and binding sites are expensive. We propose a new, purely computational method for finding putative associations between transcription factors and motifs. This method is based on a linear model that combines sequence information with expression data. We present various methods for model parameter estimation and show, via experiments on simulated data, that these methods are reliable. Finally, we examine the performance of this model on biological data and conclude that it can indeed be used to discover meaningful associations. The developed software is available as a web tool and Scilab source code at http://biit.cs.ut.ee/gmat/.

## Introduction

Regulation of gene expression is one of the most important areas of contemporary biological research. Of all the known mechanisms behind gene regulation, perhaps the most important one is the regulation of transcription by transcription factors [1], [2]. Transcription factors (*TFs*) are proteins, which bind to certain short sequences (*motifs*) in the regulatory regions (*promoters*, *enhancers*, *silencers*) of genes. This can induce or suppress the transcription of these genes into mRNA and thus affect their expression as proteins. The binding motifs for many transcription factors are not yet known and are difficult to establish by direct *in vivo* or *in vitro* experiments. Therefore, discovery of regulatory relations between the transcription factors and the genes that they regulate forms a major challenge.

In this work, we present a novel computational method for *in silico* discovery of putative associations between transcription factors and motifs from microarray gene expression and DNA sequence data. Due to overwhelming availability of this kind of data, as well as the computational simplicity of the proposed approach, our methodology can be used as a cheap and easy way to generate hypotheses concerning the networks of transcriptional regulatory control. Our experiments confirm that the generated hypotheses are biologically and statistically meaningful.

The idea to combine data about gene expression and promoter sequences for studying transcriptional regulation is not new. The main assumption behind all such methods is the premise that co-expression implies co-regulation, i.e., genes with similar gene expression profiles must be controlled by the same regulatory mechanisms [3], [4]. This assumption is most commonly exploited by clustering genes by their expression profiles [5], [6]. The promoters of co-clustered genes can then be successfully searched for overrepresented motifs using one of the multitude of motif discovery methods. We refer to [7] for a comprehensive review. This basic approach can be refined in several ways. Biclustering and other fine-grained clustering techniques allow to find gene clusters co-expressed only in certain conditions [8]. Likewise, approaches more elaborate than plain over-representation analysis might be better suited for capturing the regulatory effects within clusters, see [9], for example.

Another compelling alternative is to avoid the clustering step and reconstruct gene regulation networks by modeling expression values directly. The two major approaches here are probabilistic graphical models and predictive models. Methods of the first kind typically discretize the data to reduce the effect of noise and then find a graphical model (mainly a Bayesian network) that provides the most coherent explanation for the data [10], [11]. We refer to [12] for an excellent overview and further references.

Methods of the second kind use supervised machine learning techniques to infer a predictive model for gene expression values [13]. The resulting model needs to be easily interpretable, hence, linear models and decision trees have gained most popularity. For example, the models by [14] and [15] represent the gene mRNA expression values in a given experiment as a linear function of motif presence in the gene promoters. This allows to find motifs, the presence of which has the most influence on expression. The decision-tree based approaches by [16], [17] and [18] go a step further and predict gene expression from motif presence and transcription factor expression. As a result, these models can capture the regulatory links between transcription factors and motifs.

The G = MAT model presented in this work falls into the category of predictive models, taking its inspiration from GeneClass [17] and BDTree [16]. It is based on a special kind of a linear model that combines together expression levels of TFs and the presence of motifs in the gene promoters in order to predict mRNA levels. As a result, the coefficients of the model measure a degree of association between the transcription factors and the motifs. Hence, detecting coefficients that are statistically different from zero gives us a list of putative associations between motifs and transcription factors.

The coefficients of the model can be estimated using a variety of approaches known from classical statistics, such as least squares or regularized least squares regression [19]. In this work we present the techniques for efficient estimation of model parameters from data. We then extensively validate the reliability of our approaches in well-known yeast datasets by comparing them with other state of the art methods. The choice of yeast as a test organism is motivated by several reasons. First, the effectiveness of other methods is commonly demonstrated on few selected yeast datasets and hence we can directly compare our method to other published algorithms. Second, it is known that the main regulatory regions of yeast genes are comprised of their immediate promoters, whereas in more complex organisms the regulatory regions would often lie far away from the gene at unknown locations. Finally, as yeast is a well-studied organism, it is much easier to interpret the results. For the same reason, we use artificially generated data to experimentally study the statistical properties of our algorithms and verify that they are robust against noise. The results are encouraging on both types of data. More importantly, the method itself is not limited to yeast and can be applied to other organisms

Being a simple linear model, the method is statistically more reliable than the more complex tree-based models of GeneClass and BDTree. Additionally, it does not require data discretization and can be implemented with better efficiency. This makes G = MAT a somewhat better alternative to the former approaches. We also provide implementations of our methods in SciLab and as a Python web application (see the supplementary website) for others to test and use.

## Methods

### Basic Concepts

Although an exact definition of a gene can be argued about, here by *genes* we refer to the protein-coding regions of the DNA. More precisely, we divide genes in two non-overlapping classes: transcription factors (TFs) and target genes. The class of *transcription factors* consists of all genes that correspond to actual or putative transcription factors. The class of *target genes* (in the following referred to simply as *genes*) consists of all the remaining genes. We denote TFs by 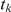, 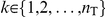 where 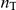 denotes the number of TFs. Similarly, we denote target genes by , 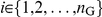 where 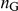 is the number of target genes. The information about which genes are transcription factors and which are not can be obtained from publicly available Gene Ontology (GO) annotation databases, such as SGD [20].

The simplest way to quantify abundance of TFs and target genes is through mRNA expression levels. These levels can be measured using a variety of microarray-based experimental techniques. Each experiment measures the expression levels of thousands, if not all, of the genes in the cell simultaneously. Typically, a single study is comprised of several microarray experiments that are collected into a single dataset. Let us denote each experiment in a study by 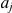, 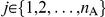, where 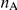 is the number of experiments. Then we can collect the expression levels of target genes into an 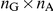 *expression matrix* 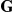 where the value 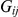 denotes the expression of a target gene 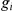 in the experiment 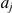. Similarly, let 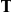 be the 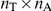 *TF expression matrix* where the value 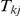 denotes the expression of the TF 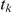 in the experiment 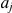.

As a second data source, we consider motif presence in promoter regions. A *motif* is a generalized representation of a binding site: a short region on the DNA, characterised by its sequence. Commonly, motifs are represented as fixed strings, strings with mismatches, position weight matrices or hidden Markov models, see [21] for further details. The exact representation type of a motif is irrelevant for our purposes, as long as we can count how many times the motif matches a promoter sequence. In the following, we denote motifs by 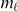, 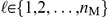 where 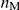 is the total number of motifs. The list of relevant motifs can consist of all possible 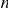-mers or can be taken form public motif transcription factor databases, such as Transfac [22], [23] or Jaspar [24].

**Figure 1:**
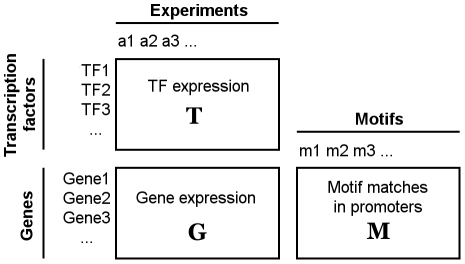
The matrices 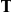
 **(top),** 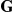 **(bottom left) and** 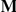 **(bottom right).** Each row of 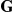 corresponds to a certain gene, as does each row of 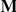. Each column of 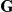 corresponds to a certain experiment, as does each column of 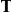. The rows of 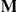 can be regarded as descriptive attributes for the rows of 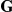, and the columns of 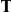 – as the attributes for the columns of 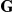.

The information about motifs in the promoters of target genes can be represented as the *motif matrix* 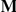, where each entry 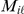 counts the number occurrences of motif 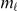 in the promoter of the target gene 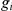. There are other ways of defining the motif matrix. For example, 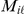 can just indicate whether a motif 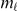 is present or not. Now the matrices 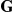, 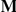 and 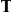 capture all the data to be analysed. Figure 1 shows a convenient way to visualize these matrices.

Although the amount of data is sufficient for statistical analysis, there are also some inherent limitations. First, our model actually quantifies the effect of transcription factors on gene expression. Therefore, ideally, we would like the matrix 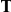 to contain *protein* expression levels of TFs, rather than their mRNA expression. Indeed, the TF *proteins* are involved in DNA binding and influence the target gene mRNA expression. However, current technology does not provide cheap methods for measuring expression levels of binding factors directly. Instead, we assume the microarray-measured mRNA expression levels to be a reasonable approximation for TF protein abundance. The assumption sweeps under the carpet the issues of translation regulation, splicing, post-translational modifications as well as the inertia of the whole process. Nonetheless, this assumption is rather common and often implicit in other similar methods [16], [17], because it is difficult to include the translation issues into the model. Luckily, mRNA expression levels are on average in good correlation with the actual protein expression levels.

Finally, it is worth mentioning that although public repositories of microarray data contain hundreds of normalized data sets, each data set having a hundred or so of microarray experiments concerning a single study, the different datasets cannot be combined easily. The differences in microarray protocols, cell cultures and laboratory conditions used in different studies make it difficult, if not impossible, to unify different datasets reliably [25].

### The G = MAT Model

In this section, we present and justify a new type of linear model for characterising gene expression. Our model is based on three simplifying assumptions about the transcriptional regulation process. Firstly, we assume that gene expression is controlled only by transcription factors. In particular, the target gene expression values in each experiment 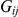 are determined by the TF expression values in the same experiments. That is, if in two experiments the expression levels of all the TFs were the same, the expression levels of all the genes would be the same, too. Thus, for every gene 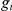 there exists some function 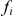 such that in experiment 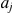:
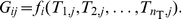
(1)


Secondly, we assume that transcription factors perform their functions by binding to certain motifs on the promoters of the target genes and the effect of each transcription factor is proportional to the number of matches of its bound motifs. Therefore, there must exist a single function 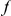 that predicts the expression level of a gene 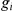 given only the expression levels of transcription factors 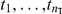 multiplied by the weights of motifs 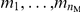 in the promoter. We can express this dependency as
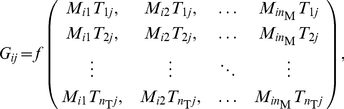
(2)where we have organised pairs 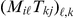 into a matrix for visual convenience.

Thirdly, we assume that we can approximate the actual prediction function 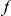 by a linear form. As a result, we obtain the G = MAT model that predicts each element of 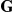 as follows:
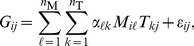
(3)where 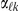 are the (unknown) model parameters and 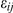 is the noise discarded by the model. Observe that the linearity in terms of pairwise products 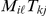 puts our model into the realm of linear models, widely studied in statistical literature. In fact, the equation (3) is also known as the *growth curve model* [26]. However, its application in the context of gene regulation is, to the best of our knowledge, entirely novel.

Now we can easily recast the equation (3) into a more compact matrix form
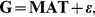
(4)where 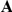 is the 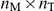 matrix of coefficients 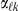. We emphasise that all the coefficients 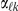 have a simple and clear interpretation. A large positive (or negative) value of 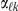 shows that expression of predictor gene is 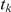 positively (or negatively) correlated with the expression of genes that have the motif 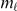 in their promoter. Similarly, a small value of 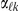 indicates that the effect of the transcription factor 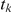 is either non-existent or highly nonlinear. Hence, a large absolute value of 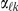 suggests that either there is a direct binding of a transcription factor to the motif 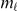, or the predictor gene 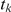 initiates a regulatory process that somehow involves the motif 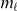.

It is important to understand that the G = MAT model is only a crude approximation of the true biological processes taking place within the cell and in practice, all the three assumptions can be violated. For instance, the gene expression is not entirely controlled by transcription factors. In reality, various other factors (such as microRNAs and environmental conditions) also influence transcriptional regulation. Neither is the effect of TFs on transcription instantaneous. Nevertheless, as long as the primary effect of TFs is significantly stronger than the other influences, we can neglect them. In particular, in the following sections, we show both theoretically and experimentally that if the unknown regulatory influence is additive and independent from the effect of TFs, then the model coefficients 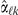 can be inferred correctly. This holds even if the amount of non-TF influence is large so that the predictive performance of the model is low.

Secondly, note that an identical motif combination in promoters does not always guarantee identical expression. Processes like DNA methylation and protein phosphorylation can interfere with binding, also the strength and location of the binding site might be of importance. Nevertheless, according to our current knowledge the second assumption is still a rather viable approximation.

The third assumption of linearity is the most questionable. We can regard the linearisation (3) as a result of the first-order Taylor approximation of the predictor function 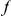. Although higher order approximations provide higher accuracy, the number of unknown parameters grows exponentially wrt model order. As a result, common parameter estimation methods become unstable or require practically infeasible amounts of microarray data. In fact, already the second-order Taylor approximation of 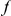 yields a model with more than 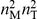 parameters and is thus practically unusable for all reasonable motif and TF counts. Of course, the linear approximation has its limitations. For instance, it cannot properly capture the combinatorial regulatory effects involving more than one TF.

Some of these secondary effects can be corrected by adding new terms into the G = MAT model. For instance, if a certain chemical compound is known to have significant impact on gene transcription, we can add its expression level to the G = MAT model as a predictor. Similarly, if a certain pair of TFs is known to act synergically, we can explicitly incorporate in the model the product of their expression values. Finally, if the expression data is a time series, we can introduce a time lag in the model by adding delayed signals as the rows of the matrix 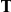.

### Parameter Estimation Methods

Next, we present a number of methods for parameter estimation for the G = MAT model. Our main emphasis is on the reliable detection of nonzero model coefficients 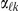, as they indicate putative relations between motifs and TFs. In the description of all parameter estimation methods we explicitly assume that matrices 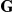, 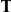 and 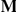 have correct dimensions. The proofs of all the results mentioned in this section are available in the supplementary text (Text S1).

#### Least Squares Regression

The most natural way of approaching the estimation problem is to search for parameter matrix 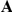, for which the mean squared error of model predictions is minimal. More formally, the *least squares fit* for the parameter matrix 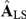 is defined as follows
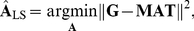
(5)where 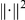 here denotes the sum of squares of the elements of a given matrix. Although the problem (5) always has a solution, sometimes the solution is not unique. To solve this ambiguity, statisticians commonly consider only the *minimum-norm* fit: a solution 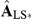 that has the least possible sum-of-squares 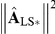.

The following two theorems describe the general solution to the problem (5) and provide sufficient and necessary conditions when the solution is unique.

**Theorem 1.** *All solutions to the problem (5) can be computed as*

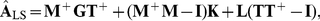
(6)
*where* 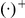 *denotes the* Moore-Penrose pseudoinverse *of a matrix*, 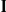 *denotes a properly-sized identity matrix and* 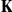 *and* 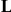 *are any two* 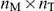 *matrices*. *The minimum norm solution to the problem* (5) *can be computed as*

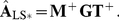
(7)


**Theorem 2.** *The problem* (5) *has a unique solution if and only if the columns of* 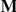 *and the rows of* 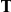 *are linearly independent, that is,* 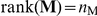 *and* 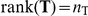 *. The corresponding solution can be computed as*

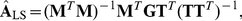
(8)
*where* 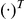 *denotes matrix transposition*.

The solution to the least squares regression problem can be computed with reasonable efficiency. Namely, the time complexity of the computation depends linearly on the number of genes 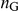 and the number of microarrays 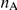, and is cubic in the number of motifs and TFs 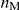 and 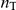. Memory requirements are linear in 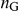 and 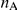, and quadratic in 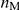 and 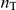. This is important, as in many practical cases the number of genes 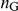 is significantly larger than 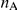, 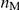 or 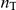.

Often, one can improve the stability of estimates by proper preprocessing of the data. The same is true for the G = MAT model. Let 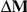 be the column-wise centered matrix 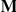, 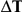 be the row-wise centered matrix 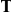, and 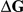 be the centered matrix 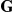. Then the corresponding minimization task
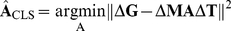
(9)gives rise to the *centered least squares* method. Informally, row- and column-wise centering of matrices 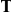 and 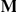 transforms the input variables of the model (3) from the form 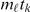 to the form 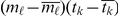. This reduces the correlations between the variables, yet preserves the correlations of the variables with the output. Consequently, the variances of the estimated coefficients for the centered G = MAT model are smaller.

In Text S1, we give a more detailed analysis and demonstrate that the centered least squares method can reliably estimate coefficients even if the dataset is incomplete, i.e., some motifs and TFs are missing, provided that the transcription factor expression values and the motif presence values are statistically independent.

#### Regularized Least Squares Regression

Least squares estimate is reliable only if the number of data points is much larger than the number of parameters. In many cases, the expression data we have does not satisfy this premise and we have to use regularization to stabilize estimates. The idea of regularization is to enforce the solution with the smallest possible parameter values by penalizing the Frobenius norm of the parameter matrix 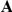. The most common regularization method is based on the 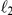 norm. The corresponding *regularized least squares fit* is defined as follows
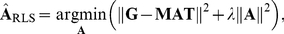
(10)where 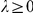 is the *regularization parameter*. Various values of 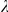 provide different trade-offs between stability and prediction accuracy. Setting 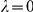 will give us the best possible prediction, but low stability for noisy data – it is just the usual least squares solution. Setting 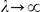 will result in a constant solution 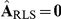, which is very stable, but useless for predicting. By choosing 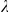 somewhere in between, we can obtain both satisfactory stability and prediction quality.

Unfortunately, the closed analytical solution for the problem (10) most probably cannot be expressed in terms of elementary algebraic operations on matrices 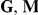 and 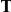 (i.e. without having to recast matrices as vectors). We therefore propose an alternative regularized solution, to which we refer as *ridge regression*

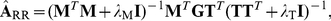
(11)where 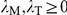 are the *regularization parameters* and 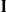 is the identity matrix. Similarly to the centered least squares, it is also possible to define *centered ridge regression* as ridge regression applied to the properly centered matrices 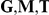.

#### Sparse Regression

Another common method of regularization is to penalize the (entry-wise) 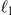-norm of the parameter matrix. This tends to produce sparse solutions (i.e., redundant parameters will be forced to zero values), hence the name of the method: *sparse regression*. The corresponding estimate is defined as follows
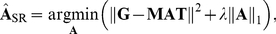
(12)where
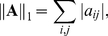
(13)and 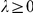 is the regularization parameter. As the solution to this problem cannot be expressed in closed form, iterative methods must be used. For example, following the *iterative thresholding* technique [27], the solution 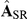 can be computed as a limit of the following sequence of iterations.
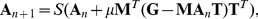
(14)where 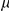 is the *step size* and 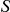 is the function which, elementwise, processes its argument as follows:
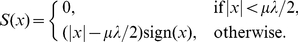
(15)


Alternatively, it is possible to show that as 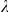 ranges from 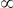 to 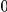 the solution 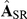 follows a piecewise-linear path, with parameters becoming nonzero one by one. It is then possible to recover the whole path as well as the order at which the parameters enter the model using the *Least Angle Regression (LARS)* algorithm [28]. The straightforward, albeit very inefficient way to perform LARS for the G = MAT model is to regard it as a linear model with 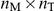 features and 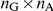 observations. The matrix structure of the model can be exploited to optimize the algorithms slightly, although the overall complexity still remains fairly high. Our implementation of *GMAT-LARS* (see Text S1) requires up to 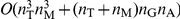 operations per iteration.

#### Correlation-based Estimate

As the set of all relevant TFs and motifs is not known and is likely to vary across different studies, a good parameter estimator method should recover coefficients 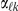 even if we have omitted some TFs and motifs from the data. The correlation based estimate derived in this subsection is ideal with this respect, since it reliably reconstructs 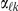 given only the data about the TF 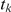 and the motif 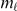, and the expression levels of all target genes. Moreover, it is possible to show that the centered least squares is in fact a good approximation to the correlation-based estimate and thus can handle missing TFs and motifs, as well. Further details are given in Text S1.

To start, note that the equation (3) can be interpreted as a a generative probabilistic model, where the measurements of all TFs in a given experiment 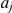 and presence of motifs in a given gene 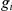 determine the gene expression level 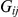. More formally, let 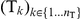 be a vector of random variables corresponding to the expression levels of TFs and 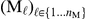 a vector of random variables corresponding to the presence of motifs. Then we can define a random variable corresponding to the gene expression level as follows
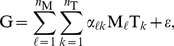
(16)where 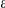 is a random error term with zero mean, independent of 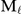 and 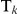 for all 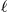 and 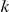.

Now, it is possible to establish connection between variable covariances and the unknown parameters 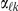 of the generative model. As usual, let 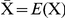 denote the mean and 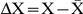 the corresponding centered variable. Let 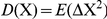 denote the variance of a random variable 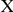. Let 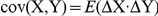 denote covariance between random variables 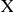 and 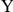. Then we can state the following theorem.

**Theorem 3.** *Assume that random variables satisfy the condition (16). If the variables* 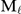 *and* 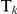 *are not constant and are pairwise independent from other random variables* 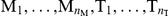 *, then*

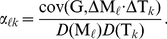
(17)


Note that the pairwise independence assumption is rather mild and is likely to be satisfied in many data sets. Hence, we can estimate 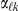, given only realizations of 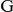, 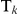 and 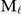. In other words, we need only the gene expression matrix 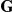, the 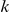-th row of 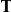, and the 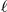-th column of 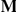. Of course, we have to replace theoretical estimates with the empirical estimates and thus the inferred coefficients 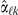 are only approximations, but the results are statistically stable.

The computation of a single coefficient with this method requires a covariance computation involving the whole matrix 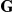, therefore, estimation of the whole matrix 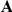 requires 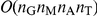 operations. It is one order of magnitude less efficient than the least squares or ridge regression estimates, but still quite tolerable for many datasets. This method lends itself easily to nearly unlimited parallelization, i.e., each coefficient can be computed independently of the others, and the covariance computation for each coefficient is highly parallelizable.

#### Randomization-based Attribute Selection

For all methods described above, we must separately decide which inferred coefficients 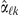 are significantly different from zero to discover putative associations between motifs and transcription factors. For that, we can compare how different are the inferred parameters 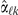 from the ones we would obtain if the gene expression values would be independent from motif and TF data. More formally, let 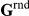 be a reordering of the matrix 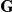 that is obtained by a random permutation of rows and columns. Let 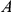 be a parameter inference method that given matrices 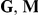 and 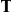 outputs an estimate for G = MAT parameters 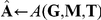. Then we can compare its behaviour using standard methods like p-values and z-scores. Here, we formalize only the z-score based attribute selection method, as other methods based on p-values have similar performance. See Text S1 for these alternative attribute selection techniques.

Let 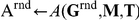 be the estimate obtained on the randomized dataset. Then we can define the *z-score* for the coefficient 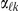 as
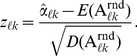
(18)


The value 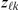 naturally measures the deviation of the true parameter estimate from the value one might obtain if the data were random.

In practice, we obtain the z-score estimate by shuffling the values of 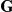 several times, computing the mean and standard deviation of each coefficient on these randomized samples and using these values to normalize the true estimate according to the equation (18). This way, for each estimated coefficient we obtain a score of how large it is in comparison to estimates, obtained on randomized data.

## Results and Discussion

### Model Performance

To demonstrate and assess the applicability of the model to biological data we first of all applied it on a dataset of yeast microarray measurements by [4]. The Spellman data is a rather popular benchmark for similar methods (e.g. [14], [16]), and it is thus possible to make comparisons. Besides, baker's yeast is a well-studied model organism, and the dataset quantifies the well-understood cell-cycle processes, which makes it easy to interpret the results. To further examine method stability, we have performed a number of tests on artificially simulated data.

#### Performance on the Spellman Dataset

The dataset by [4] consists of 77 microarray experiments measuring gene expression in the cells of the baker's yeast (*Saccharomyces cerevisiae*) at different phases of the cell cycle. We combined this data with the Transfac motif matches in the 800bp upstream genomic sequences obtained from the SGD website to get the 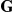, 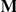 and 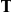 matrices for the analysis. We then applied the basic least squares estimation method on this data and considered the model coefficients with the highest (most positive) values. Table 1 lists the 5 top-scoring pairs. It is easy to see that at least three of the the five pairs obtained are indeed associated: both the F$GAL4 01 motif and the GAL1, GAL3 and GAL80 genes are related to the same family of galactokinase genes, known to be regulated by the same mechanisms [29]. It is also worth noting the considerable importance of the galactokinase genes to the cell cycle. Nothing of this kind of relevance could be obtained by the BDTree algorithm on the same data. See Text S1 for more details.

**Table 1:** G = MAT analysis of the Spellman dataset.

Motif	TF	Score
F$GAL4_01 (Binding site for GAL4)	*GAL1* (Galactokinase, phosphorylates alpha-D-galactose to alpha-D-galactose-1-phosphate in the first step of galactose catabolism.)	0.30
F$GAL4_01 (Binding site for GAL4)	*GAL3* (Transcriptional regulator involved in activation of the GAL genes in response to galactose.)	0.26
F$GAL4_01 (Binding site for GAL4)	*GAL80* (Transcriptional regulator involved in the repression of GAL genes in the absence of galactose.)	0.18
F$MCM1_02 (Binding site for MCM1 and SFF)	*SFG1* (Nuclear protein, putative transcription factor required for growth of superficial pseudohyphae (which do not invade the agar substrate) but not for invasive pseudohyphal growth.)	0.12
F$MCM1_02 (Binding site for MCM1 and SFF)	*ACE2* (Transcription factor that activates expression of early G1-specific genes, localizes to daughter cell nuclei after cytokinesis and delays G1 progression in daughters, localization is regulated by phosphorylation.)	0.12

The table presents five motif-TF pairs having the largest (most positive) values of the corresponding parameters 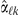. Motifs are in the leftmost column and are identified by their Transfac identifiers. The middle column contains TFs, which are identified by their gene names. The rightmost column contains the corresponding values 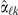.

Another strong indication in favor of the biological meaningfulness of the results was provided by a split-set experiment. If a method were overly sensitive to noise, its output would vary abruptly over different datasets even if all of them captured the same biological processes. Such behaviour would significantly reduce the practical applicability of any method. To detect such instability, we divided the Spellman dataset experiment-wise into two non-intersecting parts of 40 and 37 experiments and used our methods to find and rank TF-motif pairs for both data sets. Depending on the chosen inference parameters, the overlap between top-ten of these lists was from 3 to 4 elements – a result, which is significantly better than random (p-value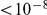). This shows a considerable statistical stability of the model – something that has not been demonstrated for most of the competing approaches.

Although the results are biologically meaningful and stable, the predictive error of the model is rather large (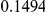), not differing much from the variance of the data (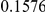). The latter can be explained by the small number of motifs used for prediction. Indeed, as we use just 38 well-known yeast motifs, we restrict the predictions for the columns of 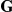 to a 38-dimensional subspace. As a result, it is almost impossible to fit column vectors with 5766 components precisely. In fact, in statistical terms, a linear model that is capable of explaining 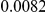 units of variance out of 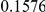 using just 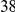 parameters out of a maximum 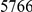, is indeed highly significant – the corresponding p-value of the F-test is 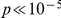.

To show that the low predictive power does not compromise the reliability of parameter estimates, we conduct a number of experiments on artificial data. These experiments convincingly demonstrate this fact, and in addition help to quantify the performance of the different parameter estimation methods.

#### Statistical Validation on Artificial Data

We generated randomly a number of datasets according to the equation (4), trying to keep the statistical characteristics of the generated data as close as possible to the Spellman dataset. Next, we attempted to estimate the matrix 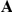 given only the matrices 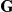, 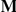, and 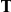 using the parameter estimation methods described previously under various perturbations of the data. We discovered that if the matrix 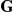 contains significant amount of additive gaussian noise and some rows/columns are missing from the matrices 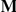 and 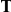, the parameters 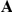 can nonetheless be estimated quite accurately. Despite the accurately estimated parameters, the predictive error of the resulting model *can nonetheless be unacceptably large* – a situation similar to the one observed in the analysis of the Spellman dataset. These results allow us to conclude that the large model error in the first experiment can be regarded as a result of noisy and incomplete data, rather than the general incorrectness of the model.

We already noted the fact, that 38 motifs are not enough to linearly explain the variance of 5766 genes. Introduction of *latent* motifs allows to theoretically “fix” the predictive performance, leaving the model parameters and their interpretation intact. Indeed, suppose that, in addition to the 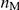 known motifs 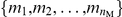, a number of other, unknown motifs 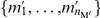 is participating in the regulation. Let 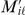 denote the presence of the unknown motif 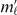 in the promoter of gene 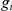 and let 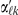 denote the regulatory interaction of motif 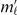 with TF 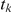. The unknown motifs can now be included into the model as follows:
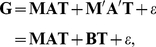
(19)where the term 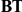 accounts for most of the noise in the original model. Despite the additional term, the parameters 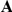 in the augmented model (19) can be estimated exactly as before. For example, the application of the least squares method with appropriate regularization penalty to the model (19) produces the same estimate (6) for 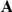 as the application of least squares to the original G = MAT model (4). The estimation of latent parameters 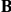 (or even 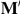 and 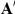) is also possible [30], [31], yet, without additional information, will only produce anonymous links between genes and TFs, which do not allow meaningful interpretation. Consult Text S1 for more details.

**Figure 2:**
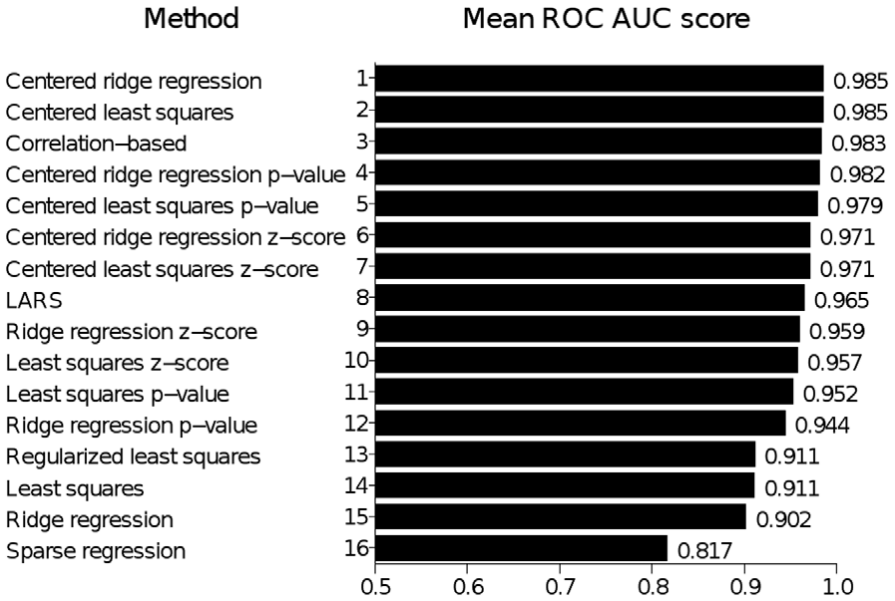
The ROC AUC score of different estimation methods, averaged over 100 runs. Note the increase in performance of the basic techniques brought by the use of randomization and a further increase due to centering. Also note the high performance of the correlation-based estimate.

Experiments on artificial data allowed us to compare the parameter estimation performance of the different methods. We generated reasonably noisy datasets, estimated the parameters using different methods, ordered the model coefficients according to their estimated values and assessed the ROC AUC score of such ordering. The resulting scores are presented in Figure 2. The conclusion from the experiments is that although all estimation methods perform rather well, the centered least squares and centered ridge regression approaches seem to show the best performance.

### Applications and Case Studies

As explained and illustrated in the previous sections, the G = MAT analysis can be used to discover putative associations between motifs and transcription factors. However, this is not the only task that can be addressed using the G = MAT model. In this section, we present a number of examples demonstrating various other applications of the G = MAT analysis in practical settings. The detailed results of all the experiments are available via the supplementary web tool.

#### Discovering Process-specific TFs and Motifs

The most obvious application for the G = MAT model is the discovery of putative TF-motif associations from gene expression and motif presence data. An example of such analysis has already been presented in section “Model Performance”. However, quite often the discovered associations are rather indirect and require extensive biological knowledge to be verified. The results are easier to interpret if we consider the top-scoring TFs and the top-scoring motifs as two separate lists. These lists contain TFs and motifs that are specific to the processes measured in the microarray data.

Such an approach was taken in the work of Middendorf et al. [17], where the authors applied their GeneClass algorithm to yeast stress response data. The GeneClass algorithm works in the same setting as the G = MAT model. Namely, it is a predictive model that uses TF-motif pairs to predict expression of target genes. Unlike the G = MAT model, the GeneClass algorithm is based on a much more complex model – an *alternative decision tree*.

The GeneClass algorithm is reported to predict expression values quite well, but its main use is the ranking of most influential TF-motif pairs. In their paper, the authors apply this algorithm to a yeast stress response dataset. They observe that the TFs and the motifs in the top-scoring pairs are indeed known to be related to stress response. We applied the G = MAT model on the same dataset and observed similar results (Table 2).

**Table 2:** G = MAT analysis of the Gasch dataset.

Motif	TF	Score
Y$GAL1_15 (Binding site for MIG1)	*USV1* (Putative transcription factor containing a C2H2 zinc finger; mutation affects transcriptional regulation of genes involved in protein folding, ATP binding, and cell wall biosynthesis.)	0.63
Y$HSP12_01 (Binding site for ABF1)	*USV1* (Putative transcription factor containing a C2H2 zinc finger; mutation affects transcriptional regulation of genes involved in protein folding, ATP binding, and cell wall biosynthesis.)	0.52
Y$HSP12_01 (Binding site for ABF1)	*RSF2* (Zinc-finger protein involved in transcriptional control of both nuclear and mitochondrial genes, many of which specify products required for glycerol-based growth, respiration, and other functions.)	0.50
Y$CHA1_04 (Binding site for ABF1)	*SHP1* (UBX (ubiquitin regulatory X) domain-containing protein that regulates Glc7p phosphatase activity and interacts with Cdc48p. SHP1 interacts with ubiquitylated proteins in vivo and is required for degradation of a ubiquitylated model substrate.)	0.50
Y$GAL1_15 (Binding site for MIG1)	*MSN1* (Transcriptional activator involved in regulation of invertase and glucoamylase expression, invasive growth and pseudohyphal differentiation, iron uptake, chromium accumulation, and response to osmotic stress; localizes to the nucleus.)	0.48

The table presents five motif-TF pairs having the largest values of the corresponding parameters 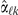. The parameter values are given in the rightmost column.

#### Data

Unfortunately, it was not possible to obtain exactly the same data as the one that was used in the GeneClass experiments due to a minor, but unrecoverable error in the supplementary materials of the GeneClass paper. However, following the instructions provided in the paper, we reconstructed a similar dataset. The dataset consists of microarray data [32] and known yeast binding sites from Transfac, matched on 500bp upstream sequences from SGD using the PATCH tool that comes with Transfac.

#### Results and comparison to GeneClass

We applied the G = MAT model on the dataset and examined the top-scoring coefficients of the model. In general, the exact ranking of the coefficients varied depending on the chosen G = MAT estimation method and its parameters. Nonetheless, a certain small set of TFs and motifs consistently occupied the top-scoring positions. This is rather similar to the situation in the GeneClass paper, where the exact ranking varied depending on the scoring algorithm, yet several TFs were consistently present in the top.

Table 2 presents the result of centered ridge regression (with 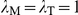), applied to the dataset. The top-scoring transcription factor, *USV1* coincides with the top-scoring regulator obtained by GeneClass. The remaining regulators differ from those reported by GeneClass, yet we believe our list to make no less sense. Indeed, the discovered TFs and motifs are known to be involved in the processes related to stress response.

- The *RSF2* gene is known to be involved in glycerol-based growth and respiration [33]. These processes have a clear relation to stress response, because use of glycerol is one of the reactions of yeast to hyperosmotic stress [34].
- The *SHP1* gene has been predicted to have a role in stress response [8].
- The *MSN1* gene is known to be involved in hyperosmotic stress [35].
- It is thought that the major function of the *MIG1* regulator is to repress the transcription of genes that are responsible for sugar utilization [36].
- The gene *ABF1* encodes a multifunctional regulator particularly involved in different chromatin-related events [37]. The highly-scoring binding site Y$HSP12_01 of this protein was originally discovered in the promoter of the *HSP12* heat shock gene [38].

Other G = MAT estimates produced different, but still meaningful results. For instance, the heat shock factor *HSF1* occupies several top-scoring positions in the G = MAT correlation-based results. As several of the microarray experiments were measuring the response of yeast to heat shock, this result makes sense.

#### Motif Discovery

So far, we used a rather small set of well-known motifs and aimed at identifying the most influential out of these. Alternatively, we can use a large set of motifs encompassing all the substrings of a given length. Finding the most influential out of that set is equivalent to identifying biologically meaningful sequences in DNA – a task known as *motif discovery*. A good overview of motif discovery methods and applications is provided in [7].

An approach similar to the G = MAT model has already been used for motif discovery in the work of Bussemaker et al. [14], where the authors applied their REDUCE algorithm for yeast promoter sequences. In brief, the idea of the REDUCE algorithm is to correlate gene expression with motif presence to score motifs and select the highest scoring ones as biologically significant. In their paper, the authors applied this idea to microarray data by [4]. Their approach was to iteratively construct a set of 7-nucleotide motifs that correlate most with the gene expression values. Conceptually, this is quite similar to what is done using the G = MAT model.

#### Data

We considered all possible 7-mers of letters {A,T,C,G} and matched them on the promoters (800bp upstream sequences) of the 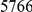 genes of the Spellman dataset. The resulting motif matrix contained 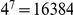 motifs, which was significantly larger than the number of genes 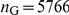 and could lead to overfitting. To reduce the number of motifs, we selected roughly 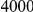 of the most frequent 7-mers (i.e. those which were present in the most promoters, there were 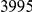 such motifs after excluding ties). The microarray dataset that we used is the one described in section “Performance on the Spellman Dataset”.

#### Results and comparison to REDUCE

The motif corresponding to the largest coefficient of the least squares estimate was AAATCTT. This does not differ much from the two top-scoring results of the REDUCE algorithm: AAAATTT and AAATTTT. Also interesting was the top-scoring motif of the G = MAT correlation-based estimate, CGATGAG. This motif is the fourth highest on the REDUCE result list. Notably, both motifs have also been discovered from the same data by various other studies [39].

#### Automatic GO Annotation

Automated assignment of relevant *Gene Ontology (GO)* annotations to genes is an important problem and a popular research direction in contemporary computational biology [40]. In this section, we demonstrate how the G = MAT model can be employed for this purpose.

In all our previous experiments, the values of model parameters could be interpreted as follows: a high 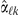 indicates that the expression of transcription factor 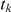 correlates well with the expression of genes that have motif 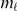 in their promoter. In this experiment, we propose to replace motifs with GO terms, and the motif matrix 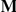 with the binary matrix of GO annotations. Formally, let 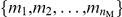 be a set of GO terms, and let
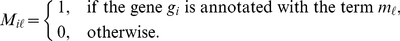
(20)


In this case, the interpretation of model parameters changes to the following: a high 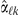 indicates that the expression of transcription factor 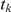 correlates well with the expression of genes that are annotated with the GO term 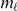. Therefore, a high value of 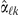 suggests that the TF 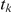 is also somehow related to the term 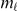. This allows to use G = MAT for discovering putative GO annotations. We illustrate the idea with an experiment.

#### Data

We used the Spellman dataset, described in section “Performance on the Spellman Dataset”, for the 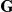 and 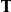 matrices. To construct the matrix 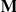, we selected 200 GO terms that had the greatest number of genes associated with them and created a 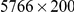 binary matrix of annotations as described above.

#### Results

Ridge regression with 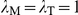, produced quite interesting results on this dataset. Out of the ten top-scoring pairs of TFs and GO terms, one corresponded to a known GO annotation. Moreover, the ten pairs with the lowest (i.e., most negative) scores contained two known annotations. The discovery of 3 true positive associations in a set of 20 predictions in this case is statistically significant (p-value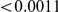). Finally, consider the five top-scoring pairs presented in Table 3. The discovered pairs are, at the very least, quite consistent. For example, the *KAR4* gene is associated to the terms “*conjugation with cellular fusion*” and “*mating projection tip*”. Both terms are related to the mating process, and the *KAR4* gene is actually known to be involved in this process. In fact, its current true annotation is “*karyogamy during conjugation with cellular fusion*”.

**Table 3:** G = MAT for GO annotation on the Spellman dataset.

Motif	TF	Score
GO:0000747 (Conjugation with cellular fusion)	*KAR4* (Transcription factor required for gene regulation in response to pheromones.)	0.07
GO:0043332 (Mating projection tip)	*KAR4* (Transcription factor required for gene regulation in response to pheromones.)	0.06
GO:0005762 (Mitochondrial large ribosomal subunit)	*RGM1* (Putative transcriptional repressor with proline-rich zinc fingers.)	0.05
GO:0006999 (Nuclear pore organization and biogenesis)	*CRF1* (Transcriptional corepressor involved in the regulation of ribosomal protein gene transcription via the TOR signaling pathway and protein kinase A, phosphorylated by activated Yak1p which promotes accumulation of Crf1p in the nucleus.)	0.05
GO:0005763 (Mitochondrial small ribosomal subunit)	*RGM1* (Putative transcriptional repressor with proline-rich zinc fingers.)	0.05

The table presents five (GO term, TF) pairs having the largest values of the corresponding parameters 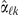.

Also, note that we can regard the obtained result as two separate lists, as we did it in section “Discovering Process-specific TFs and Motifs”. In this case, the list of top-scoring GO terms represents the important processes that were measured in the expression data.

### Conclusion

Efficient computational analysis of microarray data as well as the discovery of putative associations between transcription factors and DNA binding sites are issues of prominent importance in bioinformatics. We proposed a statistical model to address these problems. Our method can detect potential DNA-binding candidates together with the binding sites that might participate in the regulatory processes.

In particular, we studied the applicability of the model to biological data. Experiments on both real and artificial data demonstrated that our model is not predictive, but purely descriptive. That is, the prediction error of the model is very large, but the estimated parameters are still reliable and biologically meaningful. For instance, we have shown that associations discovered using our model from the well-known Spellman microarray dataset correspond to known indirect relations between transcription factors and motifs. Additionally, we illustrated how the G = MAT model can be applied in several other contexts besides the discovery of TF-motif associations. We demonstrated how the G = MAT model can be applied for the discovery of process-specific TFs and motifs, for motif discovery and for GO annotation.

## Supporting Information

Text S1 Supplementary detailed mathematical development and analysis of the method.
(0.36 MB PDF)
