# Supplementary material for: G = MAT: Linking Transcription Factor Expression and DNA Binding Data
Source: PLoS One. 2011 Jan 31;6(1):e14559. doi: 10.1371/journal.pone.0014559 (PMC3031503; doi:10.1371/journal.pone.0014559)
Supplement: Text S1 — Supplementary detailed mathematical development and analysis of the method. (0.36 MB PDF) [file pone.0014559.s001.pdf]

G=MAT: Linking Transcription Factor Expression and DNA Binding Data (Supplementary Text)

Konstantin Tretyakov[†] Sven Laur[†] Jaak Vilo[†‡*]

1 Parameter Estimation Methods

1.1 Least Squares Estimate

Theorem 1 *All solutions to the problem (5) can be computed as*

$$\hat{\mathbf{A}}_{\mathrm{LS}} = \mathbf{M}^{+}\mathbf{G}\mathbf{T}^{+} + (\mathbf{M}^{+}\mathbf{M} - \mathbf{I})\mathbf{K} + \mathbf{L}(\mathbf{T}\mathbf{T}^{+} - \mathbf{I}) \ , \tag{21}$$

where $(\cdot)^{+}$ *denotes the* Moore-Penrose pseudoinverse *of a matrix,* $\mathbf{I}$ *denotes a properly-sized identity matrix and* $\mathbf{K}$ *and* $\mathbf{L}$ *are any two* $n_{\mathrm{M}} \times n_{\mathrm{T}}$ *matrices. The minimum norm solution to the problem (5) can be computed as*

$$\hat{\mathbf{A}}_{\mathrm{LS}*} = \mathbf{M}^{+}\mathbf{G}\mathbf{T}^{+} \ . \tag{22}$$

Theorem 2 *The problem (5) has a unique solution if and only if the columns of* $\mathbf{M}$ *and the rows of* $\mathbf{T}$ *are linearly independent, that is,* $\mathrm{rank}(\mathbf{M}) = n_{\mathrm{M}}$ *and* $\mathrm{rank}(\mathbf{T}) = n_{\mathrm{T}}$*. The corresponding solution can be computed as*

$$\hat{\mathbf{A}}_{\mathrm{LS}} = (\mathbf{M}^{T}\mathbf{M})^{-1}\mathbf{M}^{T}\mathbf{G}\mathbf{T}^{T}(\mathbf{T}\mathbf{T}^{T})^{-1} \ , \tag{23}$$

where $(\cdot)^{T}$ *denotes matrix transposition.*

[†]University of Tartu, Tartu, Estonia.
[‡]Quretec Ltd, Tartu, Estonia.
[*]Corresponding author

Proof of Theorem 2. First, note that the objective function in (5) is a quadratic convex function simply because the problem is equivalent to the least squares estimation of a "traditional" linear model. Therefore, there must exist a minimum, not necessarily unique, which is achieved precisely in the point(s) where the gradient becomes $\mathbf{0}$. Hence, to find the minimum, we solve as follows:

$$\frac{\partial \|\mathbf{G} - \mathbf{MAT}\|^2}{\partial \mathbf{A}} = \mathbf{0}\,,$$
$$\frac{\partial}{\partial \mathbf{A}} \sum_{i,j} (\mathbf{G}_{ij} - (\mathbf{MAT})_{ij})^2 = \mathbf{0}\,.$$

We take the derivative of the sum of squares:

$$\sum_{i,j} 2(\mathbf{G}_{ij} - (\mathbf{MAT})_{ij}) \frac{\partial(-(\mathbf{MAT})_{ij})}{\partial \mathbf{A}} = \mathbf{0}\,,$$
$$\sum_{i,j} 2(\mathbf{G}_{ij} - (\mathbf{MAT})_{ij}) \frac{\partial\left(-\sum_{\ell,k} \alpha_{\ell k} M_{i\ell} T_{kj}\right)}{\partial \mathbf{A}} = \mathbf{0}\,,$$
$$\sum_{i,j} -2(\mathbf{G}_{ij} - (\mathbf{MAT})_{ij}) M_{i\ell} T_{kj} = 0, \text{ for all } \ell, k\,.$$

We divide the last equation by -2 and rewrite it conveniently in matrix form:

$$\sum_{i,j} (\mathbf{M}^T)_{\ell i} (\mathbf{G} - \mathbf{MAT})_{ij} (\mathbf{T}^T)_{jk} = 0, \text{ for all } \ell, k\,,$$
$$\mathbf{M}^T(\mathbf{G} - \mathbf{MAT})\mathbf{T}^T = \mathbf{0}\,,$$
$$\mathbf{M}^T\mathbf{G}\mathbf{T}^T = \mathbf{M}^T\mathbf{MATT}^T\,. \tag{24}$$

The equation has a unique solution iff the matrices $\mathbf{TT}^T$ and $\mathbf{M}^T\mathbf{M}$ are invertible, which is equivalent to the requirement $\text{rank}(\mathbf{T}) = n_\text{T}$ and $\text{rank}(\mathbf{M}) = n_\text{M}$. Multiplying the equation (24) by $(\mathbf{M}^T\mathbf{M})^{-1}$ on the left and by $(\mathbf{TT}^T)^{-1}$ on the right, we obtain the stated solution. ∎

Proof of Theorem 1, Equation (21). As we know from the proof of Theorem 2, the solutions to the problem (5) are precisely the solutions of equation (24). It remains to show that these are precisely all matrices of the form (21).

Sufficiency: Let $\hat{\mathbf{A}}_\text{LS}$ be given by equation (21). Then it is a solution of (24). To show it, we first note that, due to the properties of Moore-Penrose pseudoinverses,

$$\mathbf{M}^T\mathbf{M}(\mathbf{M}^+\mathbf{M} - \mathbf{I}) = \mathbf{M}^T(\mathbf{MM}^+\mathbf{M}) - \mathbf{M}^T\mathbf{M} = \mathbf{M}^T\mathbf{M} - \mathbf{M}^T\mathbf{M} = \mathbf{0}\,, \tag{25}$$
$$(\mathbf{TT}^+ - \mathbf{I})\mathbf{TT}^T = (\mathbf{TT}^+\mathbf{T})\mathbf{T}^T - \mathbf{TT}^T = \mathbf{TT}^T - \mathbf{TT}^T = \mathbf{0}\,, \tag{26}$$

and thus,

$$\mathbf{M}^T\mathbf{M}\hat{\mathbf{A}}_{\mathrm{LS}}\mathbf{T}\mathbf{T}^T = \mathbf{M}^T\mathbf{M}(\mathbf{M}^+\mathbf{G}\mathbf{T}^+ + (\mathbf{M}^+\mathbf{M} - \mathbf{I})\mathbf{K} + \mathbf{L}(\mathbf{T}\mathbf{T}^+ - \mathbf{I}))\mathbf{T}\mathbf{T}^T \tag{27}$$
$$= \mathbf{M}^T\mathbf{M}\mathbf{M}^+\mathbf{G}\mathbf{T}^+\mathbf{T}\mathbf{T}^T\,,$$

because after opening the brackets in (27) the second and the third term in the sum disappear due to (25) and (26). Using the Moore-Penrose pseudoinverse pseudoinverse we can now obtain:

$$\mathbf{M}^T\mathbf{M}\hat{\mathbf{A}}_{\mathrm{LS}}\mathbf{T}\mathbf{T}^T = (\mathbf{M}^T\mathbf{M}\mathbf{M}^+)\mathbf{G}(\mathbf{T}^+\mathbf{T}\mathbf{T}^T) = \mathbf{M}^T\mathbf{G}\mathbf{T}^T\,,$$

and therefore $\hat{\mathbf{A}}_{\mathrm{LS}}$ is indeed a solution to (24).

NECESSITY: Let $\mathbf{A}'$ be some solution of (24). Choose $\mathbf{K}'$ and $\mathbf{L}'$ as follows:

$$\begin{aligned}\mathbf{K}' &= (\mathbf{M}^+\mathbf{M} - \mathbf{I})(\mathbf{A}'\mathbf{T}\mathbf{T}^+ - \mathbf{M}^+\mathbf{G}\mathbf{T}^+)\\ \mathbf{L}' &= (\mathbf{A}' - \mathbf{M}^+\mathbf{G}\mathbf{T}^+)(\mathbf{T}\mathbf{T}^+ - \mathbf{I})\end{aligned}$$

And let

$$\hat{\mathbf{A}}_{\mathrm{LS}} = \mathbf{M}^+\mathbf{G}\mathbf{T}^+ + (\mathbf{M}^+\mathbf{M} - \mathbf{I})\mathbf{K}' + \mathbf{L}'(\mathbf{T}\mathbf{T}^+ - \mathbf{I})\,. \tag{28}$$

Then we can establish that

$$\hat{\mathbf{A}}_{\mathrm{LS}} = \mathbf{A}'\,.$$

To see that, simply substitute the expressions for $\mathbf{K}'$ and $\mathbf{L}'$. We do it in several steps. First, note that for any $\mathbf{X}$:

$$\begin{aligned}(\mathbf{X}^+\mathbf{X} - \mathbf{I})(\mathbf{X}^+\mathbf{X} - \mathbf{I}) &= \mathbf{X}^+\mathbf{X}\mathbf{X}^+\mathbf{X} - 2\mathbf{X}^+\mathbf{X} + \mathbf{I}\\ &= \mathbf{X}^+\mathbf{X} - 2\mathbf{X}^+\mathbf{X} + \mathbf{I} = \mathbf{I} - \mathbf{X}^+\mathbf{X}\\ (\mathbf{X}\mathbf{X}^+ - \mathbf{I})(\mathbf{X}\mathbf{X}^+ - \mathbf{I}) &= \mathbf{X}\mathbf{X}^+\mathbf{X}\mathbf{X}^+ - 2\mathbf{X}\mathbf{X}^+ + \mathbf{I}\\ &= \mathbf{X}\mathbf{X}^+ - 2\mathbf{X}\mathbf{X}^+ + \mathbf{I} = \mathbf{I} - \mathbf{X}\mathbf{X}^+\end{aligned}$$

Hence, we can expand the term $(\mathbf{M}^+\mathbf{M} - \mathbf{I})\mathbf{K}'$ as follows:

$$\begin{aligned}(\mathbf{M}^+\mathbf{M} - \mathbf{I})\mathbf{K}' &= (\mathbf{M}^+\mathbf{M} - \mathbf{I})(\mathbf{M}^+\mathbf{M} - \mathbf{I})(\mathbf{A}'\mathbf{T}\mathbf{T}^+ - \mathbf{M}^+\mathbf{G}\mathbf{T}^+)\\ &= (\mathbf{I} - \mathbf{M}^+\mathbf{M})(\mathbf{A}'\mathbf{T}\mathbf{T}^+ - \mathbf{M}^+\mathbf{G}\mathbf{T}^+)\\ &= \mathbf{A}'\mathbf{T}\mathbf{T}^+ - \mathbf{M}^+\mathbf{G}\mathbf{T}^+ - \mathbf{M}^+\mathbf{M}\mathbf{A}'\mathbf{T}\mathbf{T}^+ + \mathbf{M}^+\mathbf{M}\mathbf{M}^+\mathbf{G}\mathbf{T}^+\\ &= \mathbf{A}'\mathbf{T}\mathbf{T}^+ - \mathbf{M}^+\mathbf{G}\mathbf{T}^+ - \mathbf{M}^+\mathbf{M}\mathbf{A}'\mathbf{T}\mathbf{T}^+ + \mathbf{M}^+\mathbf{G}\mathbf{T}^+\\ &= \mathbf{A}'\mathbf{T}\mathbf{T}^+ - \mathbf{M}^+\mathbf{M}\mathbf{A}'\mathbf{T}\mathbf{T}^+ \end{aligned} \tag{29}$$

Similarly for $\mathbf{L}'(\mathbf{T}\mathbf{T}^+ - \mathbf{I})$:

$$\begin{aligned}\mathbf{L}'(\mathbf{T}\mathbf{T}^+ - \mathbf{I}) &= (\mathbf{A}' - \mathbf{M}^+\mathbf{G}\mathbf{T}^+)(\mathbf{T}\mathbf{T}^+ - \mathbf{I})(\mathbf{T}\mathbf{T}^+ - \mathbf{I})\\ &= (\mathbf{A}' - \mathbf{M}^+\mathbf{G}\mathbf{T}^+)(\mathbf{I} - \mathbf{T}\mathbf{T}^+)\\ &= \mathbf{A}' - \mathbf{M}^+\mathbf{G}\mathbf{T}^+ - \mathbf{A}'\mathbf{T}\mathbf{T}^+ + \mathbf{M}^+\mathbf{G}\mathbf{T}^+\mathbf{T}\mathbf{T}^+\\ &= \mathbf{A}' - \mathbf{M}^+\mathbf{G}\mathbf{T}^+ - \mathbf{A}'\mathbf{T}\mathbf{T}^+ + \mathbf{M}^+\mathbf{G}\mathbf{T}^+\\ &= \mathbf{A}' - \mathbf{A}'\mathbf{T}\mathbf{T}^+ \end{aligned} \tag{30}$$

Finally, substitute (29) and (30) into (28):

$$\begin{aligned}\hat{\mathbf{A}}_{\text{LS}} &= \mathbf{M}^+\mathbf{G}\mathbf{T}^+ + \mathbf{A}'\mathbf{T}\mathbf{T}^+ - \mathbf{M}^+\mathbf{M}\mathbf{A}'\mathbf{T}\mathbf{T}^+ + \mathbf{A}' - \mathbf{A}'\mathbf{T}\mathbf{T}^+ \\ &= \mathbf{A}' + \mathbf{M}^+\mathbf{G}\mathbf{T}^+ - \mathbf{M}^+\mathbf{M}\mathbf{A}'\mathbf{T}\mathbf{T}^+ . \end{aligned} \tag{31}$$

As we know, $\mathbf{A}'$ satisfies (24). Therefore, it follows that

$$\begin{gathered}\mathbf{M}^T\mathbf{G}\mathbf{T}^T = \mathbf{M}^T\mathbf{M}\mathbf{A}'\mathbf{T}\mathbf{T}^T , \\ \mathbf{M}^+(\mathbf{M}^+)^T\mathbf{M}^T\mathbf{G}\mathbf{T}^T(\mathbf{T}^+)^T\mathbf{T}^+ = \mathbf{M}^+(\mathbf{M}^+)^T\mathbf{M}^T\mathbf{M}\mathbf{A}'\mathbf{T}\mathbf{T}^T(\mathbf{T}^+)^T\mathbf{T}^+ .\end{gathered}$$

Due to the properties of Moore-Penrose pseudoinverses, this further simplifies to

$$\begin{gathered}\mathbf{M}^+\mathbf{G}\mathbf{T}^+ = \mathbf{M}^+\mathbf{M}\mathbf{A}'\mathbf{T}\mathbf{T}^+ , \\ \mathbf{M}^+\mathbf{G}\mathbf{T}^+ - \mathbf{M}^+\mathbf{M}\mathbf{A}'\mathbf{T}\mathbf{T}^+ = \mathbf{0} .\end{gathered} \tag{32}$$

Substituting (32) into (31) produces the desired result and thus completes the proof. ∎

Proof of Theorem 1, Equation (22). Let $\hat{\mathbf{A}}_{\text{LS}}$ be any solution of (5). We are going to show that

$$\left\|\hat{\mathbf{A}}_{\text{LS}}\right\|^2 = \left\|\mathbf{M}^+\mathbf{G}\mathbf{T}^+\right\|^2 + \left\|(\mathbf{M}^+\mathbf{M} - \mathbf{I})\mathbf{K} + \mathbf{L}(\mathbf{T}\mathbf{T}^+ - \mathbf{I})\right\|^2 \tag{33}$$

and therefore

$$\left\|\hat{\mathbf{A}}_{\text{LS}}\right\| \geq \left\|\mathbf{M}^+\mathbf{G}\mathbf{T}^+\right\| = \left\|\hat{\mathbf{A}}_{\text{LS}*}\right\| ,$$

from which the desired result follows.

Let

$$\mathbf{M} = \mathbf{U}_{\text{M}}\mathbf{D}_{\text{M}}\mathbf{V}_{\text{M}}^T, \qquad \mathbf{T} = \mathbf{U}_{\text{T}}\mathbf{D}_{\text{T}}\mathbf{V}_{\text{T}}^T \tag{34}$$

be the singular value decompositions of $\mathbf{M}$ and $\mathbf{T}$ respectively. Then:

$$\mathbf{M}^+ = \mathbf{V}_{\text{M}}\mathbf{D}_{\text{M}}^+\mathbf{U}_{\text{M}}^T, \qquad \mathbf{T}^+ = \mathbf{V}_{\text{T}}\mathbf{D}_{\text{T}}^+\mathbf{U}_{\text{T}}^T \tag{35}$$

Substitute the decompositions into equation (21):

$$\begin{aligned}\hat{\mathbf{A}}_{\text{LS}} &= (\mathbf{V}_{\text{M}}\mathbf{D}_{\text{M}}^+\mathbf{U}_{\text{M}}^T)\mathbf{G}(\mathbf{V}_{\text{T}}\mathbf{D}_{\text{T}}^+\mathbf{U}_{\text{T}}^T) \\ &\quad + (\mathbf{V}_{\text{M}}\mathbf{D}_{\text{M}}^+\mathbf{D}_{\text{M}}\mathbf{V}_{\text{M}}^T - \mathbf{I})\mathbf{K} + \mathbf{L}(\mathbf{U}_{\text{T}}\mathbf{D}_{\text{T}}\mathbf{D}_{\text{T}}^+\mathbf{U}_{\text{T}}^T - \mathbf{I}) \\ &= \mathbf{V}_{\text{M}}(\mathbf{D}_{\text{M}}^+\mathbf{U}_{\text{M}}^T\mathbf{G}\mathbf{V}_{\text{T}}\mathbf{D}_{\text{T}}^+)\mathbf{U}_{\text{T}}^T \\ &\quad + \mathbf{V}_{\text{M}}(\mathbf{D}_{\text{M}}^+\mathbf{D}_{\text{M}} - \mathbf{I})\mathbf{V}_{\text{M}}^T\mathbf{K} + \mathbf{L}\mathbf{U}_{\text{T}}(\mathbf{D}_{\text{T}}\mathbf{D}_{\text{T}}^+ - \mathbf{I})\mathbf{U}_{\text{T}}^T\end{aligned}$$

Due to orthogonality of $\mathbf{V}_{\text{M}}$ and $\mathbf{U}_{\text{T}}$:

$$\left\|\hat{\mathbf{A}}_{\text{LS}}\right\|^2 = \left\|\mathbf{V}_{\text{M}}^T\hat{\mathbf{A}}_{\text{LS}}\mathbf{U}_{\text{T}}\right\|^2 , \tag{36}$$

where

$$\mathbf{V}_{\mathrm{M}}^{T}\hat{\mathbf{A}}_{\mathrm{LS}}\mathbf{U}_{\mathrm{T}} = \mathbf{D}_{\mathrm{M}}^{+}\mathbf{U}_{\mathrm{M}}^{T}\mathbf{G}\mathbf{V}_{\mathrm{T}}\mathbf{D}_{\mathrm{T}}^{+} + (\mathbf{D}_{\mathrm{M}}^{+}\mathbf{D}_{\mathrm{M}} - \mathbf{I})\mathbf{V}_{\mathrm{M}}^{T}\mathbf{K}\mathbf{U}_{\mathrm{T}} + \mathbf{V}_{\mathrm{M}}^{T}\mathbf{L}\mathbf{U}_{\mathrm{T}}(\mathbf{D}_{\mathrm{T}}\mathbf{D}_{\mathrm{T}}^{+} - \mathbf{I})\,.$$

Now note, that because $\mathbf{D}_{\mathrm{M}}^{+}$ and $\mathbf{D}_{\mathrm{T}}^{+}$ are diagonal matrices

$$(\mathbf{D}_{\mathrm{M}}^{+}\mathbf{U}_{\mathrm{M}}^{T}\mathbf{G}\mathbf{V}_{\mathrm{T}}\mathbf{D}_{\mathrm{T}}^{+})_{\ell k} = (\mathbf{D}_{\mathrm{M}}^{+})_{\ell\ell}(\mathbf{U}_{\mathrm{M}}^{T}\mathbf{G}\mathbf{V}_{\mathrm{T}})_{\ell k}(\mathbf{D}_{\mathrm{T}}^{+})_{kk}\,. \tag{37}$$

This value is zero when $(\mathbf{D}_{\mathrm{M}}^{+})_{\ell\ell} = 0$ or $(\mathbf{D}_{\mathrm{T}}^{+})_{kk} = 0$.

The matrix $\mathbf{D}_{\mathrm{M}}^{+}$ is a diagonal matrix with

$$(\mathbf{D}_{\mathrm{M}}^{+})_{\ell\ell} = \begin{cases} \frac{1}{(\mathbf{D}_{\mathrm{M}})_{\ell\ell}}, & \text{if } (\mathbf{D}_{\mathrm{M}})_{\ell\ell} \neq 0, \\ 0, & \text{otherwise.} \end{cases}$$

Therefore, $\mathbf{D}_{\mathrm{M}}^{+}\mathbf{D}_{\mathrm{M}}$ is a diagonal matrix such that

$$(\mathbf{D}_{\mathrm{M}}^{+}\mathbf{D}_{\mathrm{M}})_{\ell\ell} = \begin{cases} 1, & \text{if } (\mathbf{D}_{\mathrm{M}}^{+})_{\ell\ell} \neq 0, \\ 0, & \text{otherwise.} \end{cases}$$

Thus, $(\mathbf{D}_{\mathrm{M}}^{+}\mathbf{D}_{\mathrm{M}} - \mathbf{I})$ is a diagonal matrix such that:

$$(\mathbf{D}_{\mathrm{M}}^{+}\mathbf{D}_{\mathrm{M}} - \mathbf{I})_{\ell\ell} = 0 \quad \Leftrightarrow \quad (\mathbf{D}_{\mathrm{M}}^{+})_{\ell\ell} \neq 0\,.$$

It follows that for each ℓ such that $(\mathbf{D}_{\mathrm{M}}^{+})_{\ell\ell} \neq 0$ the corresponding row of $(\mathbf{D}_{\mathrm{M}}^{+}\mathbf{D}_{\mathrm{M}} - \mathbf{I})\mathbf{V}_{\mathrm{M}}^{T}\mathbf{K}\mathbf{U}_{\mathrm{T}}$ is zero. By similar logic, for each k such that $(\mathbf{D}_{\mathrm{T}}^{+})_{kk} \neq 0$ the corresponding column of $\mathbf{V}_{\mathrm{M}}^{T}\mathbf{L}\mathbf{U}_{\mathrm{T}}(\mathbf{D}_{\mathrm{T}}\mathbf{D}_{\mathrm{T}}^{+} - \mathbf{I})$ is zero. Therefore, for each (ℓ, k), such that $(\mathbf{D}_{\mathrm{M}}^{+})_{\ell\ell} \neq 0$ and $(\mathbf{D}_{\mathrm{T}}^{+})_{kk} \neq 0$

$$((\mathbf{D}_{\mathrm{M}}^{+}\mathbf{D}_{\mathrm{M}} - \mathbf{I})\mathbf{V}_{\mathrm{M}}^{T}\mathbf{K}\mathbf{U}_{\mathrm{T}} + \mathbf{V}_{\mathrm{M}}^{T}\mathbf{L}\mathbf{U}_{\mathrm{T}}(\mathbf{D}_{\mathrm{T}}\mathbf{D}_{\mathrm{T}}^{+} - \mathbf{I}))_{\ell k} = 0\,.$$

But due to (37), these are exactly those (ℓ, k) for which $(\mathbf{D}_{\mathrm{M}}^{+}\mathbf{U}_{\mathrm{M}}^{T}\mathbf{G}\mathbf{V}_{\mathrm{T}}\mathbf{D}_{\mathrm{T}}^{+})_{\ell k}$ can be nonzero. Therefore,

$$\left\|\mathbf{V}_{\mathrm{M}}^{T}\hat{\mathbf{A}}_{\mathrm{LS}}\mathbf{U}_{\mathrm{T}}\right\|^{2} = \left\|\mathbf{D}_{\mathrm{M}}^{+}\mathbf{U}_{\mathrm{M}}^{T}\mathbf{G}\mathbf{V}_{\mathrm{T}}\mathbf{D}_{\mathrm{T}}^{+}\right\|^{2} + \left\|(\mathbf{D}_{\mathrm{M}}^{+}\mathbf{D}_{\mathrm{M}} - \mathbf{I})\mathbf{V}_{\mathrm{M}}^{T}\mathbf{K}\mathbf{U}_{\mathrm{T}} + \mathbf{V}_{\mathrm{M}}^{T}\mathbf{L}\mathbf{U}_{\mathrm{T}}(\mathbf{D}_{\mathrm{T}}\mathbf{D}_{\mathrm{T}}^{+} - \mathbf{I})\right\|^{2}\,. \tag{38}$$

Using orthogonality of $\mathbf{V}_{\mathrm{M}}$ and $\mathbf{U}_{\mathrm{T}}$ again:

$$\left\|\mathbf{D}_{\mathrm{M}}^{+}\mathbf{U}_{\mathrm{M}}^{T}\mathbf{G}\mathbf{V}_{\mathrm{T}}\mathbf{D}_{\mathrm{T}}^{+}\right\|^{2} = \left\|\mathbf{V}_{\mathrm{M}}\mathbf{D}_{\mathrm{M}}^{+}\mathbf{U}_{\mathrm{M}}^{T}\mathbf{G}\mathbf{V}_{\mathrm{T}}\mathbf{D}_{\mathrm{T}}^{+}\mathbf{U}_{\mathrm{T}}^{T}\right\|^{2} = \left\|\mathbf{M}^{+}\mathbf{G}\mathbf{T}^{+}\right\|^{2}\,, \tag{39}$$

and similarly:

$$\begin{aligned} &\left\|(\mathbf{D}_{\mathrm{M}}^{+}\mathbf{D}_{\mathrm{M}} - \mathbf{I})\mathbf{V}_{\mathrm{M}}^{T}\mathbf{K}\mathbf{U}_{\mathrm{T}} + \mathbf{V}_{\mathrm{M}}^{T}\mathbf{L}\mathbf{U}_{\mathrm{T}}(\mathbf{D}_{\mathrm{T}}\mathbf{D}_{\mathrm{T}}^{+} - \mathbf{I})\right\|^{2} \\ &= \left\|\mathbf{V}_{\mathrm{M}}((\mathbf{D}_{\mathrm{M}}^{+}\mathbf{D}_{\mathrm{M}} - \mathbf{I})\mathbf{V}_{\mathrm{M}}^{T}\mathbf{K}\mathbf{U}_{\mathrm{T}} + \mathbf{V}_{\mathrm{M}}^{T}\mathbf{L}\mathbf{U}_{\mathrm{T}}(\mathbf{D}_{\mathrm{T}}\mathbf{D}_{\mathrm{T}}^{+} - \mathbf{I}))\mathbf{U}_{\mathrm{T}}^{T}\right\|^{2} \\ &= \left\|(\mathbf{M}^{+}\mathbf{M} - \mathbf{I})\mathbf{K} + \mathbf{L}(\mathbf{T}\mathbf{T}^{+} - \mathbf{I})\right\|^{2}\,. \end{aligned} \tag{40}$$

Finally, substituting (36), (39) and (40) into (38), we obtain (33), which completes the proof. ∎

1.2 Ridge Regression

The following lemma provides some rationale and intuition for the G=MAT ridge regression estimate.

Lemma 1 (2-step linear regression) *Let* $\hat{\mathbf{A}}_{\mathrm{LS}}$ *be the minimum norm least squares fit (22) for a given dataset* $(\mathbf{G}, \mathbf{M}, \mathbf{T})$. *It is possible to compute* $\hat{\mathbf{A}}_{\mathrm{LS}}$ *in two steps:*

1. *First compute* $\hat{\mathbf{C}}_{\mathrm{LS}}$ *as a minimum norm least squares fit for the model* $\mathbf{G} = \mathbf{MC}$.

2. *Then, compute* $\hat{\mathbf{A}}_{\mathrm{LS}}$ *as a minimum norm least squares fit for the model* $\hat{\mathbf{C}}_{\mathrm{LS}} = \mathbf{AT}$.

Proof. The minimum norm least squares fit $\hat{\mathbf{C}}_{\mathrm{LS}}$ for the model $\mathbf{G} = \mathbf{MC}$ can be computed as:

$$\hat{\mathbf{C}}_{\mathrm{LS}} = \mathbf{M}^{+}\mathbf{G}\,.$$

The minimum norm least squares fit $\tilde{\mathbf{A}}_{\mathrm{LS}}$ for the model $\hat{\mathbf{C}}_{\mathrm{LS}} = \mathbf{AT}$ can be computed as:

$$\tilde{\mathbf{A}}_{\mathrm{LS}} = \hat{\mathbf{C}}_{\mathrm{LS}}\mathbf{T}^{+}\,.$$

By combining the two equations above, we see that final $\tilde{\mathbf{A}}_{\mathrm{LS}}$ is indeed equal to $\hat{\mathbf{A}}_{\mathrm{LS}}$ from equation (21):

$$\tilde{\mathbf{A}}_{\mathrm{LS}} = \hat{\mathbf{C}}_{\mathrm{LS}}\mathbf{T}^{+} = \mathbf{M}^{+}\mathbf{G}\mathbf{T}^{+} = \hat{\mathbf{A}}_{\mathrm{LS}}\,.$$

∎

The observation above shows, that the matrix **A** in G=MAT can actually be computed step by step:

- First express the gene expression values **G** as linear combinations of motifs: $\mathbf{G} = \mathbf{MC}$. This results in a $n_{\mathrm{M}} \times n_{\mathrm{A}}$ matrix **C**, that, in a sense, shows the "activity" of each motif in each experiment.

- Next, express this "motif activity" in each experiment as a linear combination of transcription factor activities: $\mathbf{C} = \mathbf{AT}$. Now the resulting **A** can be interpreted as showing the "transcription factor contribution to motif contribution to gene activity", which is exactly how we interpret **A** in G=MAT. Of course, the order of the steps can be reversed.

Now we can easily interpret the ridge-regression estimate as a two-step linear regression with ℓ_2-regularization at each step.

1.3 Sparse Regression

As it is mentioned in the main text, the optimization problem (12) has to be solved using iterative methods. In our experiments we considered two approaches: *Iterative Thresholding* [DDDM04] and *Least Angle Regression (LARS)* [EHJT04].

Both algorithms can be adapted slightly to the G=MAT model by noting that the expression $\mathbf{M}^T\mathbf{G}\mathbf{T}^T$ can be used to compute the inner products of all features with the output simultaneously (this is the analogue of the expression $\mathbf{X}^T\mathbf{y}$ in the classical linear model literature). The computational complexity of such an expression is $O((n_\mathrm{T} + n_\mathrm{M})n_\mathrm{G}n_\mathrm{A})$. This or a similar computation is typically the dominant part of every iteration in most standard sparse regression algorithms.

The LARS algorithm, in addition, requires the computation of the *equiangular vector*. On the k-th iteration the latter operation typically requires the inversion of a $k \times k$ matrix, and can therefore dominate the complexity as the number of iterations approaches the total number of features $n_\mathrm{M} \times n_\mathrm{T}$ in the model.

We refer the reader to the original papers [DDDM04, EHJT04] and to the G=MAT sample implementation on the website [Tre] for more details.

1.4 Correlation-based Estimate

Theorem 3 *Assume that random variables satisfy the condition (16). If the variables M_ℓ and T_k are not constant and are pairwise independent from other random variables $\mathsf{M}_1, \ldots, \mathsf{M}_{n_\mathrm{M}}, \mathsf{T}_1, \ldots, \mathsf{T}_{n_\mathrm{T}}$, then*

$$\alpha_{\ell k} = \frac{\mathrm{cov}(\mathsf{G}, \Delta\mathsf{M}_\ell \cdot \Delta\mathsf{T}_k)}{\mathrm{D}(\mathsf{M}_\ell)\,\mathrm{D}(\mathsf{T}_k)} \quad . \tag{41}$$

To prove this theorem we shall use the following result.

Lemma 2 *Let M_ℓ, T_k be pairwise independent random variables. Then*

$$\mathrm{cov}(\mathsf{M}_\lambda \mathsf{T}_\kappa, \Delta\mathsf{M}_\ell \cdot \Delta\mathsf{T}_k) = \begin{cases} \mathrm{D}(\mathsf{M}_\ell)\,\mathrm{D}(\mathsf{T}_k), & \text{if } \lambda = \ell, \kappa = k, \\ 0, & \text{otherwise.} \end{cases} \tag{42}$$

Proof. Using the equation $\mathrm{cov}(\mathsf{X}, \mathsf{Y}) = \mathrm{E}(\mathsf{XY}) - \mathrm{E}(\mathsf{X})\,\mathrm{E}(\mathsf{Y})$ and remembering that M_ℓ and M_λ are independent of T_k and T_κ, we compute:

$$\begin{aligned} &\mathrm{cov}(\mathsf{M}_\lambda \mathsf{T}_\kappa, \Delta\mathsf{M}_\ell \cdot \Delta\mathsf{T}_k) = \\ &\quad = \mathrm{E}(\mathsf{M}_\lambda \mathsf{T}_\kappa \Delta\mathsf{M}_\ell \Delta\mathsf{T}_k) - \mathrm{E}(\mathsf{M}_\lambda \mathsf{T}_\kappa)\,\mathrm{E}(\Delta\mathsf{M}_\ell \Delta\mathsf{T}_k) \\ &\quad = \mathrm{E}(\mathsf{M}_\lambda \Delta\mathsf{M}_\ell)\,\mathrm{E}(\mathsf{T}_\kappa \Delta\mathsf{T}_k) - \mathrm{E}(\mathsf{M}_\lambda \mathsf{T}_\kappa)\,\mathrm{E}(\Delta\mathsf{M}_\ell)\,\mathrm{E}(\Delta\mathsf{T}_k) \\ &\quad = \mathrm{E}(\mathsf{M}_\lambda \Delta\mathsf{M}_\ell)\,\mathrm{E}(\mathsf{T}_\kappa \Delta\mathsf{T}_k)\,. \end{aligned} \tag{43}$$

For $\ell \neq \lambda$, this equation evaluates to:

$$\mathrm{E}(\mathsf{M}_\lambda \Delta\mathsf{M}_\ell)\,\mathrm{E}(\mathsf{T}_\kappa \Delta\mathsf{T}_k) = \mathrm{E}(\mathsf{M}_\lambda)\,\mathrm{E}(\Delta\mathsf{M}_\ell)\,\mathrm{E}(\mathsf{T}_\kappa \Delta\mathsf{T}_k) = 0\,,$$

because $\mathrm{E}(\Delta\mathsf{M}_\ell) = 0$. Similar result holds for $k \neq \kappa$. Finally, for $\ell = \lambda$, $k = \kappa$:

$$\mathrm{E}(\mathsf{M}_\ell \Delta\mathsf{M}_\ell)\,\mathrm{E}(\mathsf{T}_k \Delta\mathsf{T}_k) = \mathrm{E}(\mathsf{M}_\ell(\mathsf{M}_\ell - \overline{\mathsf{M}_\ell}))\,\mathrm{E}(\mathsf{T}_k(\mathsf{T}_k - \overline{\mathsf{T}_k})) = \mathrm{D}(\mathsf{M}_\ell)\,\mathrm{D}(\mathsf{T}_k)\,,$$

which completes the proof. ∎

We can now prove the main theorem.

Proof of Theorem 3. By definition of random variables $\mathsf{G}, \mathsf{M}_\ell$ and T_k:

$$\mathsf{G} = \sum_{\lambda,\kappa} \alpha_{\lambda\kappa} \mathsf{M}_\lambda \mathsf{T}_\kappa + \varepsilon\,.$$

Therefore,

$$\mathrm{cov}(\mathsf{G}, \Delta\mathsf{M}_\ell \cdot \Delta\mathsf{T}_k) = \sum_{\lambda,\kappa} \alpha_{\lambda\kappa}\,\mathrm{cov}(\mathsf{M}_\lambda \mathsf{T}_\kappa, \Delta\mathsf{M}_\ell \cdot \Delta\mathsf{T}_k) + \mathrm{cov}(\varepsilon, \Delta\mathsf{M}_\ell \cdot \Delta\mathsf{T}_k)\,, \quad (44)$$

because of the linearity of the covariance operation. The last term in the sum (44) is zero:

$$\mathrm{cov}(\varepsilon, \Delta\mathsf{M}_\ell \cdot \Delta\mathsf{T}_k) = 0\,,$$

because ε is independent of M_ℓ and T_k.

All the remaining terms can be evaluated using Lemma 2. It follows that the only nonzero term in the sum (44) is the one where $\lambda = \ell$ and $\kappa = k$. The whole equation now becomes

$$\mathrm{cov}(\mathsf{G}, \Delta\mathsf{M}_\ell \cdot \Delta\mathsf{T}_k) = \alpha_{\ell k}\,\mathrm{D}(\mathsf{M}_\ell)\,\mathrm{D}(\mathsf{T}_k)\,,$$

from which the result follows. ∎

1.5 Centered Least Squares

It turns out, that it is possible to compute a good approximation to the correlation-based estimate efficiently using the familiar least-squares technique. Let $(\mathsf{G}, \mathsf{M}_\ell, \mathsf{T}_k)$ be random variables that satisfy the conditions of Theorem 3. Let $(\mathbf{G}, \mathbf{M}, \mathbf{T})$ be a G=MAT dataset obtained as a sample of these variables. In the following we prove that if we apply the least squares method to the *centered* versions of matrices $\mathbf{G}$, $\mathbf{M}$ and $\mathbf{T}$, we shall obtain consistent estimates for $\alpha_{\ell k}$.

In the following, let $\overline{\mathbf{M}^{(*\ell)}}$ denote the average of the values in the ℓ-th column of $\mathbf{M}$, let $\overline{\mathbf{T}^{(k*)}}$ denote the average of the k-th row of $\mathbf{T}$ and let $\overline{\mathbf{G}}$ denote the average of all elements of $\mathbf{G}$. Let $\mathbf{\Delta M}$ be the column-wise centered version of the matrix $\mathbf{M}$, $\mathbf{\Delta T}$ be the row-wise centered version of the matrix $\mathbf{T}$, and $\mathbf{\Delta G}$ be the centered version of the matrix $\mathbf{G}$. That is,

$$(\mathbf{\Delta M})_{i\ell} = M_{i\ell} - \overline{\mathbf{M}^{(*\ell)}}\,, \quad (\mathbf{\Delta T})_{kj} = T_{kj} - \overline{\mathbf{T}^{(k*)}}\,, \quad (\mathbf{\Delta G})_{ij} = G_{ij} - \overline{\mathbf{G}}\,.$$

Finally, let $\hat{\mathbf{A}}_{\mathrm{CLS}}$ be the least-squares estimate for the model:

$$\hat{\mathbf{G}}' = \mathbf{\Delta M A \Delta T}\,.$$

Then the following result holds.

Theorem 4 (Centered least squares) *Let $(\mathsf{G}, \mathsf{M}_\ell, \mathsf{T}_k)$ be random variables, satisfying the conditions of Theorem 3. Let $(\mathbf{G}, \mathbf{M}, \mathbf{T})$ be a sample, obtained from these variables and let $\hat{\mathbf{A}}_{\mathrm{CLS}}$ be obtained as described above. Then as the sample size increases, i.e., $n_{\mathrm{G}}, n_{\mathrm{A}} \to \infty$, the elements of the matrix $\hat{\mathbf{A}}_{\mathrm{CLS}}$ converge almost surely to the true model parameters $(\alpha_{\ell k})$.*

Proof. First, note that in the process $n_{\mathrm{G}} \to \infty$ the matrix $\frac{1}{n_{\mathrm{G}}}\mathbf{\Delta M}^T\mathbf{\Delta M}$ converges to the matrix of motif covariances. Indeed,

$$\left(\frac{1}{n_{\mathrm{G}}}\mathbf{\Delta M}^T\mathbf{\Delta M}\right)_{\ell\lambda} = \frac{1}{n_{\mathrm{G}}}\sum_i (M_{i\ell} - \overline{\mathbf{M}^{(*\ell)}})(M_{i\lambda} - \overline{\mathbf{M}^{(*\lambda)}})\,,$$

which, by strong law of large numbers, converges almost surely to $\mathrm{cov}(\mathsf{M}_\ell, \mathsf{M}_\lambda)$, and therefore

$$\left(\frac{1}{n_{\mathrm{G}}}\mathbf{\Delta M}^T\mathbf{\Delta M}\right)_{\ell\lambda} \overset{\text{a.s.}}{\to} \mathrm{cov}(\mathsf{M}_\ell, \mathsf{M}_\lambda) = \begin{cases} \mathrm{D}(\mathsf{M}_\ell), & \text{if } \ell = \lambda, \\ 0, & \text{otherwise.} \end{cases} \tag{45}$$

Similarly, in the process $n_{\mathrm{A}} \to \infty$:

$$\left(\frac{1}{n_{\mathrm{A}}}\mathbf{\Delta T}\mathbf{\Delta T}^T\right)_{k\kappa} \overset{\text{a.s.}}{\to} \mathrm{cov}(\mathsf{T}_k, \mathsf{T}_\kappa) = \begin{cases} \mathrm{D}(\mathsf{T}_k), & \text{if } k = \kappa, \\ 0, & \text{otherwise.} \end{cases} \tag{46}$$

Thus, the matrices $\mathbf{\Delta M}^T\mathbf{\Delta M}$ and $\mathbf{\Delta T}\mathbf{\Delta T}^T$ converge with probability 1 to diagonal matrices with nonzero elements on the diagonal. The latter matrices are invertible and therefore, for sufficiently large n_{G} and n_{A}, the matrices $\mathbf{\Delta M}^T\mathbf{\Delta M}$ and $\mathbf{\Delta T}\mathbf{\Delta T}^T$ are also invertible, because the determinants of those matrices must converge to nonzero values. Hence, for sufficiently large n_{G} and n_{A} the least squares estimate $\hat{\mathbf{A}}_{\mathrm{CLS}}$ can be computed using equation (23):

$$\begin{aligned} \hat{\mathbf{A}}_{\mathrm{CLS}} &= (\mathbf{\Delta M}^T\mathbf{\Delta M})^{-1}\mathbf{\Delta M}^T\mathbf{\Delta G}\mathbf{\Delta T}^T(\mathbf{\Delta T}\mathbf{\Delta T}^T)^{-1} \\ &= \left(\frac{1}{n_{\mathrm{G}}}\mathbf{\Delta M}^T\mathbf{\Delta M}\right)^{-1}\left(\frac{1}{n_{\mathrm{G}}n_{\mathrm{A}}}\mathbf{\Delta M}^T\mathbf{\Delta G}\mathbf{\Delta T}^T\right)\left(\frac{1}{n_{\mathrm{A}}}\mathbf{\Delta T}\mathbf{\Delta T}^T\right)^{-1}. \end{aligned} \tag{47}$$

Finally, the matrix

$$\left(\frac{1}{n_{\mathrm{G}}n_{\mathrm{A}}}\mathbf{\Delta M}^T\mathbf{\Delta G}\mathbf{\Delta T}^T\right)_{\ell k} = \frac{1}{n_{\mathrm{G}}n_{\mathrm{A}}}\sum_{i,j}(G_{ij} - \overline{\mathbf{G}})(M_{i\ell} - \overline{\mathbf{M}^{(*\ell)}})(T_{kj} - \overline{\mathbf{T}^{(k*)}})\,,$$

must also obey the law of large numbers:

$$\left(\frac{1}{n_{\mathrm{G}}n_{\mathrm{A}}}\mathbf{\Delta M}^T\mathbf{\Delta G}\mathbf{\Delta T}^T\right)_{\ell k} \stackrel{\text{a.s.}}{\to} \mathrm{cov}(\mathsf{G}, (\mathsf{M}_\ell - \overline{\mathsf{M}_\ell})(\mathsf{T}_k - \overline{\mathsf{T}_k}))\,. \tag{48}$$

The equations (45), (46) and (48) demonstrate that all terms on the right side of the equation (47) converge. Therefore, $\hat{\mathbf{A}}_{\mathrm{CLS}}$ must converge too:

$$(\hat{\mathbf{A}}_{\mathrm{CLS}})_{\ell k} \stackrel{\text{a.s.}}{\to} \frac{1}{\mathsf{D}(\mathsf{M}_\ell)} \cdot \mathrm{cov}(\mathsf{G}, (\mathsf{M}_\ell - \overline{\mathsf{M}_\ell})(\mathsf{T}_k - \overline{\mathsf{T}_k})) \cdot \frac{1}{\mathsf{D}(\mathsf{T}_k)} = \alpha_{\ell k}$$

by Theorem 3. ∎

1.6 Randomization-based Attribute Selection

In the main text, we proposed a z-score-based method for identifying the most "interesting" coefficients. Analogously to estimating z-scores for the coefficients, we might measure their *p-values*:

$$p_{\ell k} = \Pr[\mathsf{A}^{\mathrm{rnd}}_{\ell k} > \hat{\alpha}_{\ell k}]\,. \tag{49}$$

A low p-value indicates that obtaining value larger than $\hat{\alpha}_{\ell k}$ is rather unprobable and thus the corresponding coefficient is significant.

Another useful nonparametric test is obtained by estimating the sampling distribution of the coefficients using a resampling technique like bootstrap. In particular, we propose to measure the *positivity weight* of the attribute $\alpha_{\ell k}$ as the probability that its estimated value will be greater than zero:

$$w_{\ell k} = \Pr[\mathsf{A}_{\ell k} > 0]\,. \tag{50}$$

In our experiments we use the ridge-regression estimation method for the function $A(\cdot)$, because it is the most efficient of the available strategies.

2 Model Performance Analysis

In order to assess the model's performance we applied it to several artificial and real-life datasets. As we are mainly concerned here with the question of whether the model is, in general, useful for dealing with biological data rather than the specific results, we limit our presentation with a single detailed case-study here. The results of model evaluation on the other datasets (yeast stress response, human lung fibroblast transcription profiling) are similar in spirit. These can be accessed from the supplementary website.

2.1 The Spellman Dataset

For the first application of our method on real biological data we consider the microarray dataset of Spellman et al. [SSZ+98]. The dataset contains 77 microarray experiments, measuring the expression of 6178 yeast genes at different points of the cell life cycle.

Data preparation Some preprocessing was required to obtain the matrices $\mathbf{G}$ and $\mathbf{T}$.

- The dataset contained missing values. We imputed them using the *KNN-impute* algorithm [TCS+01] implemented in Matlab.
- Of all the genes present in the dataset, 318 had the *transcription regulator activity* GO annotation (according to the data of the *SGD* project [SGD]). We selected these 318 genes as transcription factors. The expression values of these TFs were collected in the 318×77 matrix $\mathbf{T}$.
- From other 5860 genes we selected 5766 for which the genomic sequence was available via the *SGD* website. The expression values of these genes were collected in the 5766×77 matrix $\mathbf{G}$.

Next, we prepared the motif matrix $\mathbf{M}$ as follows.

- For each target gene we downloaded its promoter region: 800-nucleotide-long genomic sequence upstream of the gene's translation start site. We obtained the data from the *SGD* website.
- We used the TRANSFAC database of regulatory motifs and selected the 36 known yeast motifs there.
- We used the `storm` [SSZ07] tool to match TRANSFAC motifs on the promoter. As a result we obtained the 5766×36 binary matrix $\mathbf{M}$.

G=MAT analysis. Once the data was in the form of the $\mathbf{G}$, $\mathbf{M}$ and $\mathbf{T}$ matrices, we were free to apply any of the G=MAT parameter estimation methods. We chose to apply the least-squares estimate (22), because this method is the most straightforward and requires setting no parameters. Recall that each

coefficient $\hat{\alpha}_{\ell k}$ of the resulting parameter matrix $\hat{\mathbf{A}}_{\mathrm{LS}*}$ measures a putative association between motif m_ℓ and TF t_k. Table 1 presents the pairs of motifs and TFs that correspond to the 5 coefficients of the matrix $\hat{\mathbf{A}}_{\mathrm{LS}*}$ with the largest values.

**Motif**	**TF**	**Score**
F$GAL4_01<br>Binding site for GAL4.	*GAL1*<br>Galactokinase, phosphorylates alpha-D-galactose to alpha-D-galactose-1-phosphate in the first step of galactose catabolism.	0.30
F$GAL4_01<br>Binding site for GAL4.	*GAL3*<br>Transcriptional regulator involved in activation of the GAL genes in response to galactose.	0.26
F$GAL4_01<br>Binding site for GAL4.	*GAL80*<br>Transcriptional regulator involved in the repression of GAL genes in the absence of galactose.	0.18
F$MCM1_02<br>Binding site for MCM1 and SFF.	*SFG1*<br>Nuclear protein, putative transcription factor required for growth of superficial pseudohyphae (which do not invade the agar substrate) but not for invasive pseudohyphal growth.	0.12
F$MCM1_02<br>Binding site for MCM1 and SFF.	*ACE2*<br>Transcription factor that activates expression of early G1-specific genes, localizes to daughter cell nuclei after cytokinesis and delays G1 progression in daughters, localization is regulated by phosphorylation.	0.12

Table 1: G=MAT analysis of the Spellman dataset. The table presents five motif-TF pairs having the largest values of the corresponding parameters $\hat{\alpha}_{\ell k}$. Motifs are in the leftmost column and are identified by their TRANSFAC identifiers. The middle column contains TFs, which are identified by their gene names. The rightmost column contains the corresponding values $\hat{\alpha}_{\ell k}$.

2.2 Evaluation of Results

As we have chosen a true biological dataset for the experiment, we can not know the corresponding "true" parameter matrix $\mathbf{A}$. As a result, we lack any gold standard for objectively assessing the relevance of the obtained results and have to limit ourselves with the following options:

- evaluating the biological meaningfulness of the results manually,
- evaluating the generalization ability using a split-set experiment,

- examining the predictive performance of the model.

The results evaluations seem to differ radically.

Biological relevance of the results. The top-scoring motif-TF pairs, presented in Table 1, make complete sense. In particular, the top three entries associate the known binding site of the Gal4p transcription factor with the Gal1p, Gal3p and Gal80p proteins. This is in perfect accordance with current biological knowledge [LVZ95].

- Overexpression of galactokinase-coding gene *GAL1* results in activation of Gal4p protein [BOH90, BH92]. This process is probably the reason for strong positive association between *GAL1* expression and presence of the Gal4p binding site: high expression of *GAL1* induces expression of Gal4p, which binds to that motif.

- *GAL3* is a gene highly similar to *GAL1* [PRHR00]. The corresponding protein Gal3p forms a complex with Gal80p and thus relieves inhibition of Gal4p [LVZ95]. This explains the positive association of *GAL3* with the Gal4p binding motif.

- Gal80p is a transcriptional inhibitor of GAL genes (in particular, *GAL4*). Inhibition is relieved by Gal1p or Gal3p binding [TRR02]. Therefore, we might expect Gal80p to be strongly *negatively* associated with the Gal4p binding site: the higher the expression of Gal80p, the more repressed is Gal4p, the less effect it has on the genes with the corresponding motif in the promoter. Our analysis, on the contrary, indicates a positive association. This might be due to the complex regulatory feedback loops involved in the regulation of the whole family of GAL genes. Such nonlinear relations cannot be accounted for by our simple linear model. Nonetheless, we consider the discovered relation a success rather than a failure of the analysis.

It is very unlikely that this result (three obviously successful pairs among the top five) could be obtained by chance. To verify that, we considered all motif-TF pairs, where the TF belonged to the same family of proteins as the binding factor for the motif. For example, in the above case, *GAL1*, *GAL3* and *GAL80* all belong to the same family as *GAL4* which is the binding factor for motif F$GAL4_01. There were 67 such matching pairs among the 36×318 coefficients in the parameter matrix. If we were to pick 5 pairs at random, we would expect less than 0.03 hits on average. Getting 3 hits instead exceeds the expectations more than 100-fold.

Generalization ability. In order to test the generalization ability of the model we have performed a split-set experiment on the Spellman data. Namely, the whole dataset of 77 experiments was divided into two nonintersecting parts consisting of the first 40 and the last 37 experiments respectively. Applying

the G=MAT inference methods on these two datasets resulted in top-ten lists of pairs that typically contained between 3 and 4 common TF-motif pairs, depending on the chosen parameters. This can be regarded as an indication that the model does generalize well on biological data. Indeed, the p-value of this result, i.e. the probability of obtaining two top-ten lists with 4 common elements from a "random" algorithm, is less than 10^{-10}.

Predictive performance. We could expect that if our model parameters have biological relevance and are reproducible in split-set experiments, the model should predict well. Unfortunately, it turns out that the predictive performance of the constructed model is very poor. The coefficient of determination of the model is barely above 0.05, see below.

Mean squared error:	0.1494
Variance of $\mathbf{G}$:	0.1576
Coefficient of determination, R^2:	0.0520

It might seem surprising, that a model which produces biologically meaningful results predicts so badly. However, this is mostly a flaw of intuition – the model is in fact highly significant. Indeed, note that in terms of motifs we have a linear model that is capable of explaining 0.0082 units of variance out of 0.1576 using just 38 parameters out of a maximum 5766. This corresponds to an F-value of

$$F = \frac{0.0082/37}{0.1576/5728} \approx 8.06$$

which is highly significant (p-value $\ll 10^{-5}$ according to the $F(37, 5728)$ distribution). To further alleviate any doubts, in the following we study this issue on an artificially generated dataset.

2.3 The Artificial Dataset

To study the properties of G=MAT estimates, we need a dataset that is similar to a real one yet for which we know the true value of $\mathbf{A}$. To construct such a dataset, we first studied the statistical properties of the Spellman dataset considered in the previous sections. We then randomly generated matrices $\mathbf{M}$, $\mathbf{A}$ and $\mathbf{T}$ to obtain a dataset with similar properties.

We generated a dataset with 100 transcription factors, 100 motifs and 1000 genes.

The motif matrix M. The matrix $\mathbf{M}$ is a binary matrix of motif occurrences. As a natural simplification, we regard each column of this matrix as a set of i.i.d. realizations of Bernoulli-distributed random variables. That is:

$$M_{i\ell} \sim \mathrm{B}(p_\ell)\,,$$

where p_ℓ is the probability of motif m_ℓ presence in a randomly chosen gene.

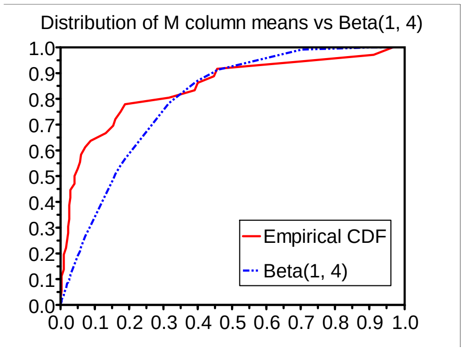

Figure 1: The empirical cumulative distribution function of motif presence probabilities p_ℓ, estimated on the Spellman dataset, compared to the cdf of the Beta$(1, 4)$ distribution.

By analyzing the Spellman dataset we see that the average absolute correlation of the columns of the matrix $\mathbf{M}$ is less than 0.02. We can therefore safely assume the columns to be independent. Finally, we observe that the distribution of probabilities p_ℓ can be reasonably well described by a Beta$(1, 4)$ distribution, see Figure 1. Therefore, we generate the artificial matrix $\mathbf{M}$ as follows:

$$p_\ell \leftarrow \text{Beta}(1, 4) \text{ independently for each column } \ell,$$
$$M_{i\ell} \leftarrow \text{B}(p_\ell) \text{ independently for each row } i.$$

The TF expression matrix T. The matrix $\mathbf{T}$ of the Spellman dataset is more difficult to model than the motif matrix. Visual inspection shows no clear column or row-based structure. The mean absolute correlation of matrix rows is 0.16, and of the columns: 0.14. We chose to model $\mathbf{T}$ as a set of independent columns. Even though it does not correspond exactly to what we observe on the Spellman dataset, the assumption of independent microarray experiments is quite plausible.

The distribution of values in each column of $\mathbf{T}$ can be very well described by normal distribution (see Figure 2). Examination of the means and standard deviations of different $\mathbf{T}$ columns shows that these can also be reasonably well described by normal distributions (Figure 3). Therefore, we generate the artificial matrix $\mathbf{T}$ as follows:

$$m_j \leftarrow \text{N}(0, 0.08^2) \text{ independently for each column } j,$$
$$\sigma_j \leftarrow \text{N}(0.35, 0.09^2) \text{ independently for each column } j,$$
$$T_{kj} \leftarrow \text{N}(m_j, \sigma_j^2) \text{ independently for each row } k.$$

The parameter matrix A. We generate matrix $\mathbf{A}$ as follows: first set all values to zero, then choose 3% of the coefficients randomly and set their values

to 0.05. The reason for the choice of such a strategy is the following. Firstly, we believe the true $\mathbf{A}$ to contain a very small number of nonzero elements. Secondly, we wish the distribution of values in $\mathbf{T}$ to be reasonably similar to the distribution of values in $\mathbf{G}$ in terms of mean and variance. Experiments showed that this can be achieved by setting the nonzero values of $\mathbf{A}$ to 0.05.

The gene expression matrix G. The matrix $\mathbf{G}$ is generated according to the model $\mathbf{G} = \mathbf{MAT}$. In some experiments we also add noise to it, but we discuss this further in the text.

2.4 Prediction versus Attribute Selection

As we saw in Section 2.2, a G=MAT model can discover relevant parameters without achieving a satisfactory predictive performance. In this section, we explain this phenomenon by demonstrating both experimentally and theoretically how and why this can happen. We start with an artificial dataset introduced in the previous section and consider two factors that influence model prediction error: noise and incomplete data. We show that although these factors can significantly decrease the model's predictive ability, they do not prevent the model from discovering relevant parameters. In the following we refer to this capability as *attribute selection*.

2.4.1 Experimental Setup

Let $\mathbf{G}^{\mathrm{a}}, \mathbf{M}^{\mathrm{a}}, \mathbf{A}^{\mathrm{a}}$ and $\mathbf{T}^{\mathrm{a}}$ denote the matrices of the artificial dataset, generated as described Section 2.3. In the experiments that follow, we introduce certain modifications to these matrices (such as add noise to $\mathbf{G}^{\mathrm{a}}$ or drop rows from $\mathbf{T}^{\mathrm{a}}$), estimate the parameter matrix $\hat{\mathbf{A}}$ using different methods and examine the performance of the estimated models in prediction and attribute selection. We measure this performance as follows.

Predictive performance. To measure predictive performance we simply consider the mean squared error of the model:

$$\mathrm{MSE}(\hat{\mathbf{A}}) = \frac{1}{n_{\mathrm{G}} \times n_{\mathrm{A}}} \left\| \mathbf{G}^{\mathrm{a}} - \mathbf{M}^{\mathrm{a}} \hat{\mathbf{A}} \mathbf{T}^{\mathrm{a}} \right\|^2 .$$

Attribute selection performance. To measure how well the estimated matrix can be used for attribute selection we use the *ROC area under curve (ROC AUC)* statistic. We consider those model parameters for which the true value $\alpha^{\mathrm{a}}_{\ell k}$ was nonzero (in other words, parameters with values 0.05), as *positives*, and all the rest as *negatives*. We then sort the parameters according to their estimated values $\hat{\alpha}_{\ell k}$ and evaluate the ROC AUC of this sorted list. If the positives all turn out to be on top of the list (i.e., their values estimated as the largest), the value of ROC AUC will be 1. This means the model is perfect for attribute selection. The ROC AUC score of 0.5 indicates a model that is not better than

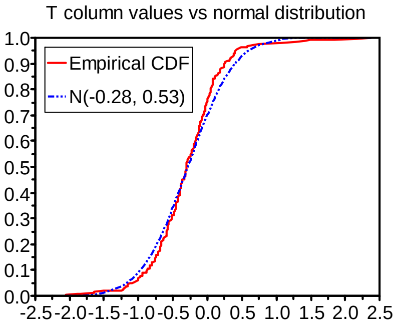

Figure 2: The empirical cumulative distribution of values in the second column of $\mathbf{T}$ can be approximated well by a Gaussian distribution $\mathrm{N}(-0.28, 0.53)$. The situation is similar for other columns.

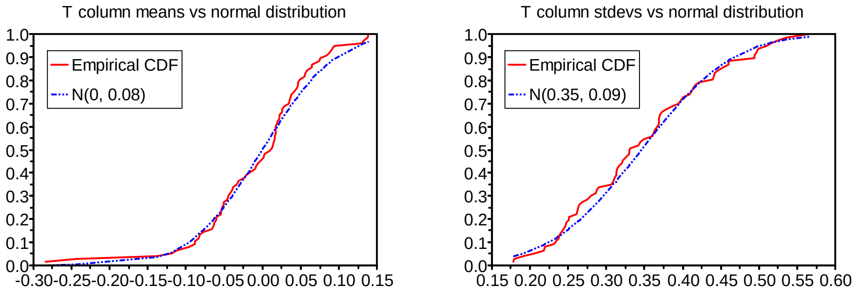

Figure 3: The empirical cumulative distribution of the means (left) and standard deviations (right) of $\mathbf{T}$ columns can be well approximated by a Gaussian distribution.

random in guessing the relevant parameters. Finally, the ROC AUC score is 0 if all the positives end up on the bottom of the list. This corresponds to a model that is good for attribute selection, but somewhy orders attributes in reverse.

2.4.2 Influence of Noise

Consider the following modification to the matrix $\mathbf{G}^{\mathrm{a}}$ of the artificial dataset:

$$\mathbf{G}^{\mathrm{a}} = \mathbf{M}^{\mathrm{a}}\mathbf{A}^{\mathrm{a}}\mathbf{T}^{\mathrm{a}} + \varepsilon\,, \tag{51}$$

where ε is Gaussian noise with mean 0 and variance σ_ε^2. This corresponds to incorporating into the data a multitude of independent factors that cannot be accounted for by the model parameters: external influences, nonlinear expression regulation rules, measurement errors, etc. Let us examine how the prediction and attribute selection performance depends on σ_ε^2. For that we created 11 artificial datasets, differing only in the variance of the noise σ_ε^2, and applied the least squares method and the ridge regression method with parameters $\lambda_{\mathrm{M}} = \lambda_{\mathrm{T}} = 100$ to these datasets.

Effect on prediction error. When the data is noise-free, i.e., $\sigma_\varepsilon^2 = 0$, the least squares method will restore $\mathbf{A}^{\mathrm{a}}$ precisely and the prediction error will be zero. When $\sigma_\varepsilon^2 > 0$, the least squares estimate is not able to account for most of the noise and the predictive error is proportional to σ_ε^2. Other methods, such as ridge regression, behave similarly. Their prediction error, by definition, is always greater or equal than that of the least squares estimate. Experiment results are presented in Figure 4 (left).

Effect on attribute selection. Figure 4 (right) shows how ROC AUC score depends on the noise. As we see, the ROC AUC of the least squares estimate deteriorates quite quickly with the introduction of noise. This is a natural result of *overfitting* that takes place here due to the improperly large number of parameters of the model. If the number of TFs n_{T} was less than the number of arrays n_{A}, the ROC AUC of the least squares estimate would deteriorate much slower. We illustrate this by considering an artificial dataset with only 50 transcription factors instead of 100, the results are presented in Figure 5. Note that in both cases the ridge regression estimate avoids overfitting and has a high ROC AUC score despite the noise.

2.4.3 Influence of Incomplete Data

Another important factor that can influence the predictive performance of the model in practice is the incompleteness of data. Let us now generate the noise-free version of the artificial dataset $(\mathbf{G}^{\mathrm{a}}, \mathbf{M}^{\mathrm{a}}, \mathbf{A}^{\mathrm{a}}, \mathbf{T}^{\mathrm{a}})$ and then drop the first n rows of $\mathbf{T}^{\mathrm{a}}$ and the first n columns of $\mathbf{M}^{\mathrm{a}}$. In some sense, this is similar to adding noise to $\mathbf{G}^{\mathrm{a}}$, but conceptually this is somewhat different. This time we say that our model is true, we just do not have enough data.

Effect on prediction error. When $n = 0$ the prediction error is 0 and when $n = 100$ the prediction error equals $\|\mathbf{G}^{\mathrm{a}}\|^2$, increasing rather uniformly in between, see Figure 6 (left).

Effect on attribute selection. Figure 6 shows that when we remove some TFs and motifs from the dataset, thus fitting a model with a smaller number of parameters than needed to explain the data completely, the ROC AUC score still stays satisfactorily high.

2.4.4 Theoretical Justification

In addition to the obvious experimental evidence, we provide an elegant theoretical justification, demonstrating how the model can estimate relevant parameters from incomplete data without being able to predict well. Let us return once more to the example with the Spellman dataset from Section 2.2.

Motifs are the bottleneck. The major bottleneck for model performance on the Spellman dataset was actually the small number of motifs. Indeed, if the number of motifs n_{M} were greater or equal to the number of genes n_{G}, model error would necessarily be 0. In our case, however, the number of motifs $n_{\mathrm{M}} = 36$ is significantly smaller than the number of genes $n_{\mathrm{G}} = 5766$. It follows from basic linear algebra, that an arbitrary vector of dimension 5766 can not in general be represented as a linear combination of just 36 base vectors. Therefore, also the G=MAT model error must be high.

To illustrate this more formally, we consider the following model:

$$\hat{\mathbf{G}} = \mathbf{MC}\,, \tag{52}$$

where $\mathbf{C}$ is an $n_{\mathrm{M}} \times n_{\mathrm{A}}$ matrix of parameters. First, note that this model is better than the G=MAT model in terms of prediction.

Theorem 5 *Let $\mathbf{G}, \mathbf{M}, \mathbf{T}$ be G=MAT matrices. Let $\hat{\mathbf{C}}_{\mathrm{LS}}$ be the least squares fit for the model (52) and let*

$$\mathrm{MSE}_{\mathrm{GMC}}(\hat{\mathbf{C}}_{\mathrm{LS}}) = \frac{1}{n_{\mathrm{G}} \times n_{\mathrm{A}}} \left\| \mathbf{G} - \mathbf{M}\hat{\mathbf{C}}_{\mathrm{LS}} \right\|^2$$

be the corresponding model error. Let $\hat{\mathbf{A}}_{\mathrm{LS}}$ be the G-MAT least squares fit and let

$$\mathrm{MSE}_{\mathrm{GMAT}}(\hat{\mathbf{A}}_{\mathrm{LS}}) = \frac{1}{n_{\mathrm{G}} \times n_{\mathrm{A}}} \left\| \mathbf{G} - \mathbf{M}\hat{\mathbf{A}}_{\mathrm{LS}}\mathbf{T} \right\|^2$$

be the corresponding model error. Then

$$\mathrm{MSE}_{\mathrm{GMC}}(\hat{\mathbf{C}}_{\mathrm{LS}}) \le \mathrm{MSE}_{\mathrm{GMAT}}(\hat{\mathbf{A}}_{\mathrm{LS}})\,.$$

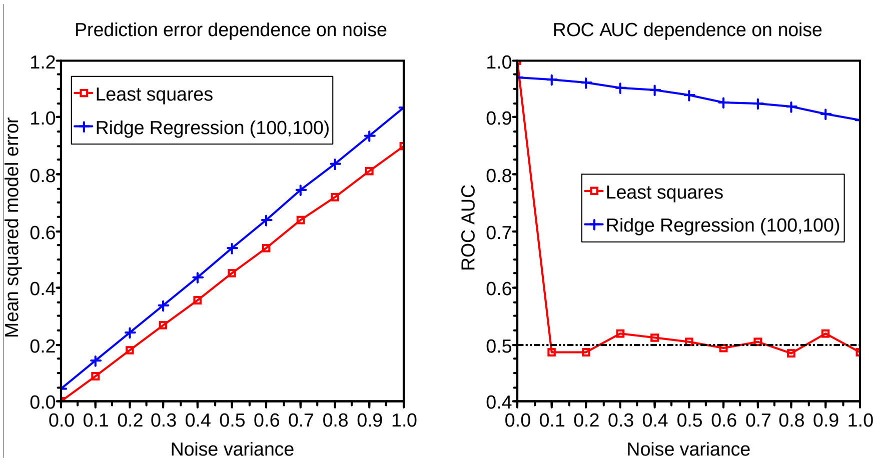

Figure 4: Influence of noise on prediction error and ROC AUC. The left plot demonstrates that the mean squared error of the least squares and ridge regression estimates is proportional to the variance of the noise. The right plot shows that the ROC AUC score of the least squares estimate deteriorates rapidly with the introduction of noise due to overfitting, but the ridge regression estimate stays resistant to noise.

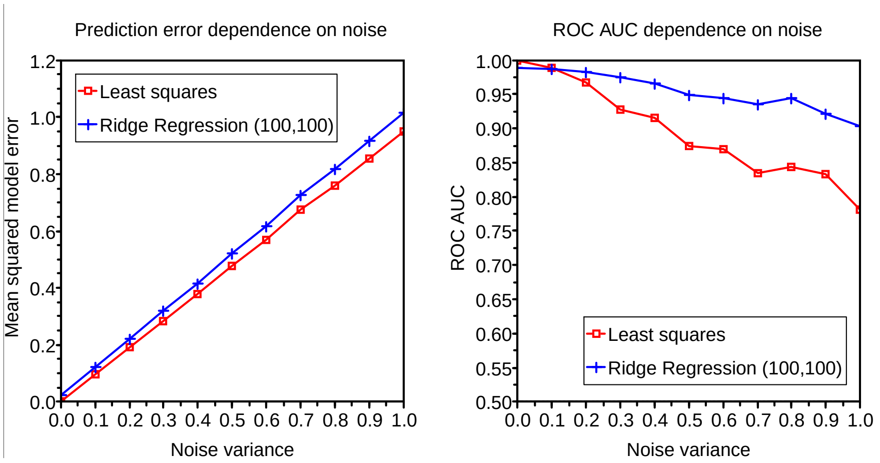

Figure 5: Influence of noise on prediction error and ROC AUC. Here we used an artificial dataset with only 50 transcription factors rather than 100. Unlike the situation in Figure 4, there is no overfitting here and the least squares estimate can tolerate the noise well. Nevertheless, the ridge regression estimate is still more stable.

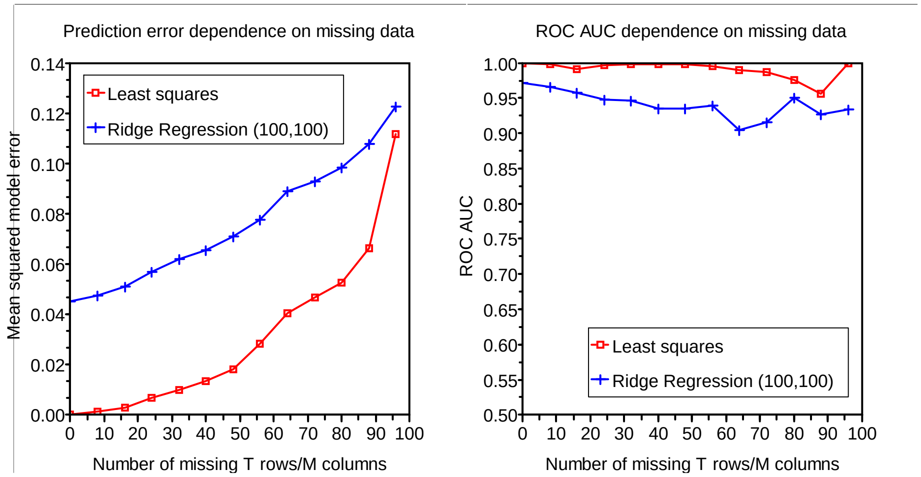

Figure 6: Influence of missing data on prediction error and ROC AUC. If we drop columns from $\mathbf{T}^{\mathrm{a}}$ and rows from $\mathbf{M}^{\mathrm{a}}$, thus hiding some TFs and motifs from the dataset, the model error increases (left) but the ROC AUC stays high (right).

Proof. Let $\hat{\mathbf{C}}_* = \hat{\mathbf{A}}_{\mathrm{LS}}\mathbf{T}$. The error of the G=MC model for such value of $\mathbf{C}$ is then equal to

$$\begin{aligned}\mathrm{MSE}_{\mathrm{GMC}}(\hat{\mathbf{C}}_*) &= \frac{1}{n_{\mathrm{G}} \times n_{\mathrm{A}}} \left\| \mathbf{G} - \mathbf{M}\hat{\mathbf{C}}_* \right\|^2 \\ &= \frac{1}{n_{\mathrm{G}} \times n_{\mathrm{A}}} \left\| \mathbf{G} - \mathbf{M}\hat{\mathbf{A}}_{\mathrm{LS}}\mathbf{T} \right\|^2 = \mathrm{MSE}_{\mathrm{GMAT}}(\hat{\mathbf{A}}_{\mathrm{LS}})\end{aligned}$$

By definition, $\hat{\mathbf{C}}_{\mathrm{LS}}$ is the matrix of model parameters for which the model error is minimal and therefore

$$\mathrm{MSE}_{\mathrm{GMC}}(\hat{\mathbf{C}}_{\mathrm{LS}}) \leq \mathrm{MSE}_{\mathrm{GMC}}(\hat{\mathbf{C}}_*) = \mathrm{MSE}_{\mathrm{GMAT}}(\hat{\mathbf{A}}_{\mathrm{LS}})\,.$$

∎

Next, note that in the G=MC model (52) each column of $\mathbf{G}$ is represented as a linear combination of the columns of $\mathbf{M}$, with coefficients given in the corresponding column of $\mathbf{C}$. As we have noted above, this is rather improbable that there exists an exact representation of the 5766-dimensional column of $\mathbf{G}$ in terms of just 36 columns of $\mathbf{M}$ and therefore it is not surprising that with high probability there does not exist a $\mathbf{C}$ for which the error of the G=MC model would be low on the Spellman dataset. As it follows from the above theorem, the error of the G=MAT model must be at least as high as that of the G=MC model. Evaluation on data shows that, in fact, the error of the G=MC model on the Spellman dataset is exactly equal to the error of the G=MAT model. This explains why the bottleneck lies precisely in the low number of motifs: it is just not possible to predict better using a linear model with so few motifs.

Introducing latent motifs. We could improve the predictive performance of the model if we added additional motifs $m_{n_\mathrm{M}+1}, m_{n_\mathrm{M}+2}, m_{n_\mathrm{M}+3}, \ldots, m_{n_\mathrm{G}}$ to the data. Suppose that these motifs do exist and let the unknown motif occurrence matrix for these new motifs be $\mathbf{M}_\mathrm{new}$. The full motif matrix $\mathbf{M}_\mathrm{full}$ is then the concatenation of the matrices $\mathbf{M}$ and $\mathbf{M}_\mathrm{new}$:

$$\mathbf{M}_\mathrm{full} = \left(\mathbf{M} \;\; \mathbf{M}_\mathrm{new}\right).$$

We also incorporate new rows into the parameter matrix to account for the new motifs. The full parameter matrix $\mathbf{A}_\mathrm{full}$ is therefore:

$$\mathbf{A}_\mathrm{full} = \begin{pmatrix} \mathbf{A} \\ \mathbf{A}_\mathrm{new} \end{pmatrix}.$$

The G=MAT model in the presence of the new motifs now takes the following form:

$$\begin{aligned} \hat{\mathbf{G}} &= \mathbf{M}_\mathrm{full}\mathbf{A}_\mathrm{full}\mathbf{T} = \\ &= (\mathbf{MA} + \mathbf{M}_\mathrm{new}\mathbf{A}_\mathrm{new})\mathbf{T} = \\ &= \mathbf{MAT} + \mathbf{M}_\mathrm{new}\mathbf{A}_\mathrm{new}\mathbf{T} = \mathbf{MAT} + \mathbf{BT}, \end{aligned} \tag{53}$$

where $\mathbf{B}$ is the additional matrix of parameters that needs to be estimated.

It is natural to estimate the parameters $(\mathbf{A}, \mathbf{B})$ of the model (53) using the least squares method with a penalty on $\mathbf{B}$.

Definition 1 *Define the* least squares fit *for the parameter matrices* $(\hat{\mathbf{A}}_\mathrm{LS}, \hat{\mathbf{B}}_\mathrm{LS})$ *of the model (53) as follows:*

$$(\hat{\mathbf{A}}_\mathrm{LS}, \hat{\mathbf{B}}_\mathrm{LS}) = \operatorname*{argmin}_{\mathbf{A},\mathbf{B}} \|\mathbf{G} - \mathbf{MAT} - \mathbf{BT}\|^2 + \lambda \|\mathbf{B}\|^2 , \tag{54}$$

where $\lambda > 0$ *is the penalty coefficient.*

Quite surprisingly, the solution $\hat{\mathbf{A}}_\mathrm{LS}$ to (54) is *exactly* equal to the least squares solution of G=MAT (21).

Theorem 6 *The solution to the problem (54) can be computed as follows:*

$$\hat{\mathbf{A}}_\mathrm{LS} = \mathbf{M}^+\mathbf{G}\mathbf{T}^+ + (\mathbf{M}^+\mathbf{M} - \mathbf{I})\mathbf{K} + \mathbf{L}(\mathbf{T}\mathbf{T}^+ - \mathbf{I}), \tag{55}$$

$$\hat{\mathbf{B}}_\mathrm{LS} = (\mathbf{G} - \mathbf{M}\hat{\mathbf{A}}_\mathrm{LS}\mathbf{T})\mathbf{T}^T(\mathbf{T}\mathbf{T}^T + \lambda\mathbf{I})^{-1}, \tag{56}$$

where $\mathbf{K}$ *and* $\mathbf{L}$ *are any* $n_\mathrm{M} \times n_\mathrm{T}$ *matrices.*

Proof. The proof is similar to the proof of Theorem 2. The objective function in problem (54) is a convex quadratic function and to find its minimum we search for the points where the gradient is zero:

$$\frac{\partial \|\mathbf{G} - \mathbf{MAT} - \mathbf{BT}\|^2 + \lambda \|\mathbf{B}\|^2}{\partial \mathbf{A}} = \mathbf{0}, \tag{57}$$

$$\frac{\partial \|\mathbf{G} - \mathbf{MAT} - \mathbf{BT}\|^2 + \lambda \|\mathbf{B}\|^2}{\partial \mathbf{B}} = \mathbf{0}. \tag{58}$$

We start with equation (57) and solve as in Theorem 2:

$$\frac{\partial}{\partial \mathbf{A}} \sum_{i,j} (\mathbf{G}_{ij} - (\mathbf{MAT})_{ij} - (\mathbf{BT})_{ij})^2 = \mathbf{0}$$

$$\sum_{i,j} 2(\mathbf{G}_{ij} - (\mathbf{MAT})_{ij} - (\mathbf{BT})_{ij}) \frac{\partial(-(\mathbf{MAT})_{ij})}{\partial \mathbf{A}} = \mathbf{0}\,,$$

$$\sum_{i,j} -2(\mathbf{G}_{ij} - (\mathbf{MAT})_{ij} - (\mathbf{BT})_{ij}) M_{i\ell} T_{kj} = 0, \text{ for all } \ell, k\,,$$

$$\mathbf{M}^T(\mathbf{G} - \mathbf{MAT} - \mathbf{BT})\mathbf{T}^T = \mathbf{0}\,. \tag{59}$$

Next, we proceed with equation (58) similarly:

$$\frac{\partial}{\partial \mathbf{B}} \sum_{i,j} (\mathbf{G}_{ij} - (\mathbf{MAT})_{ij} - (\mathbf{BT})_{ij})^2 + \frac{\partial}{\partial \mathbf{B}} \lambda \sum_{i,k} B_{ik}^2 = \mathbf{0}$$

$$\sum_{i,j} 2(\mathbf{G}_{ij} - (\mathbf{MAT})_{ij} - (\mathbf{BT})_{ij}) \frac{\partial(-(\mathbf{BT})_{ij})}{\partial \mathbf{B}} + 2\lambda \mathbf{B} = \mathbf{0}\,, \tag{60}$$

Consider the derivative of $-(\mathbf{BT})_{ij}$ with respect to $B_{\iota\kappa}$:

$$\frac{\partial(-(\mathbf{BT})_{ij})}{\partial B_{\iota\kappa}} = \frac{\partial\left(-\sum_k B_{ik} T_{kj}\right)}{\partial B_{\iota\kappa}} = \begin{cases} -T_{\kappa j}, & \text{if } i = \iota, \\ 0, & \text{otherwise.} \end{cases} \tag{61}$$

Substituting it into (60) gives

$$\sum_j -2(\mathbf{G}_{\iota j} - (\mathbf{MAT})_{\iota j} - (\mathbf{BT})_{\iota j}) T_{\kappa j} + 2\lambda B_{\iota\kappa} = 0, \text{ for any } \iota, \kappa\,,$$

which can be rearranged to the matrix form:

$$(\mathbf{G} - \mathbf{MAT} - \mathbf{BT})\mathbf{T}^T = \lambda \mathbf{B}. \tag{62}$$

Now substitute (62) into (59):

$$\mathbf{M}^T(\mathbf{G} - \mathbf{MAT} - \mathbf{BT})\mathbf{T}^T = \lambda \mathbf{M}^T \mathbf{B} = \mathbf{0}\,,$$

and thus, because $\lambda \neq 0$:

$$\mathbf{M}^T \mathbf{B} = \mathbf{0}\,.$$

As a result, we can transform equation (59) to the familiar form:

$$\begin{aligned} \mathbf{M}^T(\mathbf{G} - \mathbf{MAT} - \mathbf{BT})\mathbf{T}^T &= \mathbf{M}^T(\mathbf{G} - \mathbf{MAT})\mathbf{T}^T - \mathbf{M}^T\mathbf{BTT}^T \\ &= \mathbf{M}^T(\mathbf{G} - \mathbf{MAT})\mathbf{T}^T - \mathbf{0} \\ &= \mathbf{M}^T(\mathbf{G} - \mathbf{MAT})\mathbf{T}^T = \mathbf{0}\,. \end{aligned}$$

The solutions to this equation are exactly the solutions of (24) given by Theorem 1:

$$\hat{\mathbf{A}}_{\mathrm{LS}} = \mathbf{M}^+\mathbf{GT}^+ + (\mathbf{M}^+\mathbf{M} - \mathbf{I})\mathbf{K} + \mathbf{L}(\mathbf{TT}^+ - \mathbf{I})\,. \tag{63}$$

Finally, substituting (63) into (59) we get:

$$
\begin{aligned}
(\mathbf{G} - \mathbf{M}\hat{\mathbf{A}}_{\mathrm{LS}}\mathbf{T} - \mathbf{B}\mathbf{T})\mathbf{T}^T &= \lambda \mathbf{B}, \\
(\mathbf{G} - \mathbf{M}\hat{\mathbf{A}}_{\mathrm{LS}}\mathbf{T})\mathbf{T}^T - \mathbf{B}\mathbf{T}\mathbf{T}^T &= \lambda \mathbf{B}, \\
(\mathbf{G} - \mathbf{M}\hat{\mathbf{A}}_{\mathrm{LS}}\mathbf{T})\mathbf{T}^T &= \mathbf{B}\mathbf{T}\mathbf{T}^T + \lambda \mathbf{B}, \\
(\mathbf{G} - \mathbf{M}\hat{\mathbf{A}}_{\mathrm{LS}}\mathbf{T})\mathbf{T}^T &= \mathbf{B}(\mathbf{T}\mathbf{T}^T + \lambda \mathbf{I}).
\end{aligned}
$$

The matrix $(\mathbf{T}\mathbf{T}^T + \lambda\mathbf{I})$ is always invertible for $\lambda > 0$, and therefore, the solution $\hat{\mathbf{B}}_{\mathrm{LS}}$ is:

$$
\hat{\mathbf{B}}_{\mathrm{LS}} = (\mathbf{G} - \mathbf{M}\hat{\mathbf{A}}_{\mathrm{LS}}\mathbf{T})\mathbf{T}^T(\mathbf{T}\mathbf{T}^T + \lambda\mathbf{I})^{-1}\,.
$$

∎

The result of Theorem 6 means that by introducing a number of unknown (*latent*) motifs into G=MAT, we still keep the value and interpretation of the parameter matrix $\hat{\mathbf{A}}_{\mathrm{LS}}$ at the same time elegantly getting rid of the "motif bottleneck". The predictive error of the new model (53) is significantly lower. For example, on the Spellman dataset the G=MAT+BT model has a mean squared error of 0.

It should be noted, that there exist approaches, typically known as *Network Component Analysis (NCA)* methods, which attempt to produce meaningful fits to latent features [KYB+04, ND06]. However, such fits are only possible when certain additional information is available, such as ChIP-chip binding data. In addition, there is no obvious way to link latent features to actual motifs, i.e. to split the estimate of $\mathbf{B}$ to a product $\mathbf{M}_{\mathrm{new}}\mathbf{A}_{\mathrm{new}}$.

To summarize, we have presented both experimental and theoretical evidence for the possibility of our model to perform well for attribute selection despite the very high mean squared error.

2.5 Comparison of Methods

Finally, we use the artificial dataset to compare the attribute selection performance of the different G=MAT estimation methods.

2.6 On the choice of the λ Parameter

A number of methods require setting the regularization parameter λ. To perform a fair comparison, we evaluate the performance of these methods for several values of λ on a single dataset.

Test dataset. We constructed the test dataset as follows. First, we generated a noise-free dataset $(\mathbf{G}^{\mathrm{a}}, \mathbf{M}^{\mathrm{a}}, \mathbf{A}^{\mathrm{a}}, \mathbf{T}^{\mathrm{a}})$ as described in Section 2.3. Let $\sigma^2 = \mathrm{D}(\mathbf{G}^{\mathrm{a}})$ be the variance of $\mathbf{G}^{\mathrm{a}}$. We added zero-mean Gaussian noise to $\mathbf{G}^{\mathrm{a}}$ with variance $4\sigma^2$. Finally, we dropped 80 rows of $\mathbf{T}^{\mathrm{a}}$ and 80 columns of $\mathbf{M}^{\mathrm{a}}$ thus leaving only 20 TFs and 20 motifs in the dataset. We believe that such a noisy setup with a significant lack of information might correspond closely to the real biological situation.

Methods. In the experiment we compared the following G=MAT methods: regularized least squares, ridge regression with $\lambda_M = \lambda_T = \lambda$, sparse regression (implemented using iterative thresholding), p-value-based attribute selector for ridge regression, z-score-based attribute selector for ridge regression and positivity-based attribute selector for ridge regression. For each method and for each value of the regularization parameter λ we computed the ROC AUC score as described above.

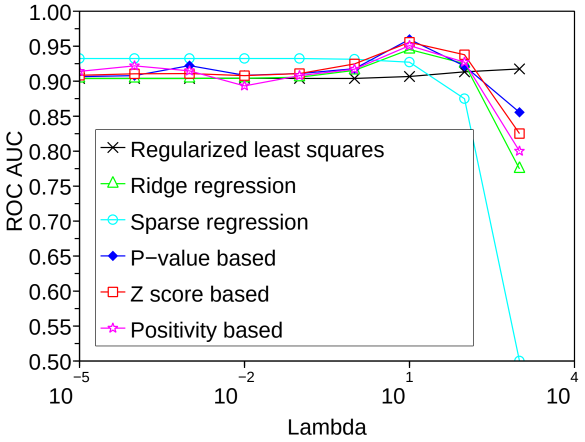

Figure 7: The ROC AUC score of different estimation methods for different values of λ.

Results. The results are depicted in Figure 7. We can clearly see that there was no significant difference in performance among all methods on this dataset – for all methods λ could be rather freely chosen within the range $0 \ldots 10$ with little effect on performance: the relatively small number of model parameters prevents overfitting and hence there is no real need for regularization.

Choice of λ on real data. It must be noted that in the experiments on real data (the Spellman dataset) the choice of regularization parameters within a wide range does not seem to have a significant effect on the ordering of the parameters either. For example, the set of top ten parameters computed using ridge regression with $\lambda_M = \lambda_T = 0.01$ is exactly the same as the set of top ten parameters computed using $\lambda_M = \lambda_T = 1$, and has 5 common elements with the set of top ten parameters computed using $\lambda_M = \lambda_T = 100$.

2.7 Comparison of Performance

In the previous section, we compared the parameterized algorithms on a single dataset and discovered that most of them perform well when $\lambda = 10$. Here, we fix this value of λ for all parameterized methods. This way we get rid of the parameterization issue, and can perform a fair comparison among all methods.

Test setup. We test each method 100 times by generating a new random dataset on every iteration. Each dataset is generated as described in the previous section. The ROC AUC scores of all runs are averaged to produce the final score.

Methods. We add the following parameter-free methods into the comparison: least squares regression, correlation-based estimate, p-value, z-score and positivity based attribute selectors for least squares, and the LARS algorithm. When assessing the output of LARS, instead of regarding actual estimated parameter values, we score parameters by the order in which they are introduced into the model during LARS iterations. This parameter ordering is then used in the ROC computation.

Results. The results are presented in Figure 8. In general, all methods perform quite well, most of them achieving ROC AUC score higher than 0.9. The best performance (0.983) is achieved by the correlation-based estimate. Although it differs only slightly from the second best result (LARS, 0.965), the difference is statistically significant. In 76 iterations out of 100 the correlation-based estimate was better than the one based on LARS. The results also clearly demonstrate how the p-value and z-score-based randomization techniques improve the performance of the base methods.

It it notable, that the sparse regression estimate based on iterative thresholding is outperformed by the more conventional linear regression techniques. This might be in part due to the abundant gaussian noise in the test dataset, which misleads the ℓ_1 penalty stronger than the more accomodating ℓ_2-regularization. Note that the high performance of the LARS method can be explained by the fact that in order to apply LARS the data must be centered. As we will see in the following, centering significantly improves the performance of *all* G=MAT estimation methods. In the following we avoid the use of sparse estimates because they require extra computational power but do not offer significant benefits.

The Effect of Centering. The correlation-based estimate, despite its good performance on our test dataset, can be computationally expensive. In Theorem 4 we have demonstrated that an approximation can be obtained by applying the least squares method to the properly centered data. Here, we demonstrate that this is indeed the case. Figure 9 introduces the centered least squares and centered ridge regression into comparison as well as their p-value and z-score randomizations. We see that the centered versions of the least squares and ridge regression perform even better than the correlation-based approach,

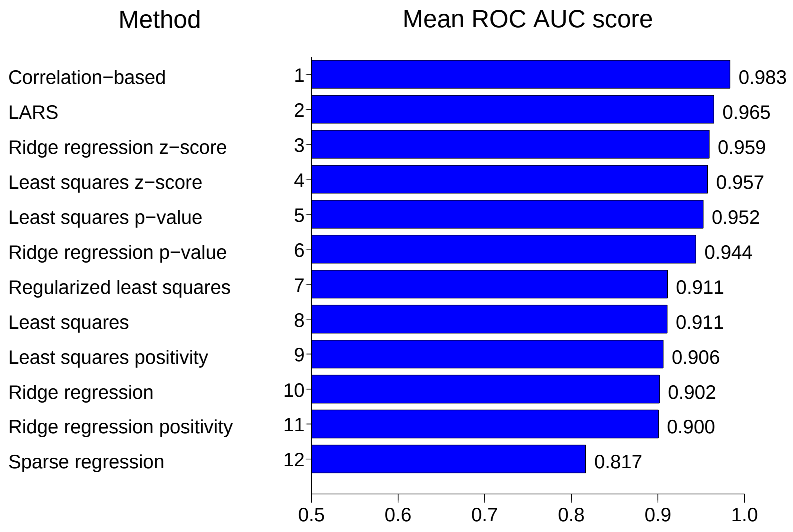

Figure 8: The ROC AUC score of different estimation methods, averaged over 100 runs.

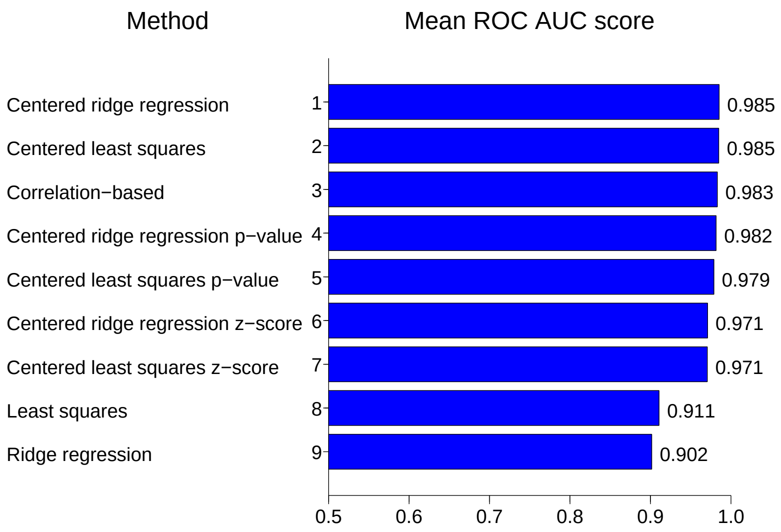

Figure 9: The ROC AUC score of different estimation methods, averaged over 100 runs.

thus beating all other methods. It is worth noting that the p-value and z-score randomizations could not further boost their performance.

References

[BH92] P. J. Bhat and J. E. Hopper. Overproduction of the GAL1 or GAL3 protein causes galactose-independent activation of the GAL4 protein: evidence for a new model of induction for the yeast GAL/MEL regulon. *Mol Cell Biol*, 12(6):2701–2707, Jun 1992.

[BOH90] P. J. Bhat, D. Oh, and J. E. Hopper. Analysis of the GAL3 signal transduction pathway activating GAL4 protein-dependent transcription in Saccharomyces cerevisiae. *Genetics*, 125(2):281–291, Jun 1990.

[DDDM04] Ingrid Daubechies, Michel Defrise, and Christine De Mol. An iterative thresholding algorithm for linear inverse problems with a sparsity constraint. *Communciation Pure Application*, LVII:1413–1457, 2004.

[EHJT04] Bradley Efron, Trevor Hastie, Iain Johnstone, and Robert Tibshirani. Least angle regression. *Ann. Statist.*, 32(2):407–499, 2004.

[KYB+04] Katy C Kao, Young-Lyeol Yang, Riccardo Boscolo, Chiara Sabatti, Vwani Roychowdhury, and James C Liao. Transcriptome-based determination of multiple transcription regulator activities in escherichia coli by using network component analysis. *Proc Natl Acad Sci U S A*, 101(2):641–646, Jan 2004. Authors apply ”network component analysis”, which is essentially a decomposition of the expression matrix E as E=CT where T are TF activities and C is the control signal matrix.

[LVZ95] D. Lohr, P. Venkov, and J. Zlatanova. Transcriptional regulation in the yeast GAL gene family: a complex genetic network. *FASEB J*, 9(9):777–787, Jun 1995.

[ND06] Dat H Nguyen and Patrik D’haeseleer. Deciphering principles of transcription regulation in eukaryotic genomes. *Mol Syst Biol*, 2:2006.0012, 2006. First does NCA (See MANetworker, Kao2004, Boulesteix2005) to decompose expression as E=MA. Then splits gene set into ”gene ensembles” by motif presence, which is further split by promoter features into smaller sets, which allows to learn a model ”promoter features, motif” -¿ ”motif strength”, which can then be used for predictions. Authors claim better performance on CV than REDUCE.

[PRHR00] A. Platt, H. C. Ross, S. Hankin, and R. J. Reece. The insertion of two amino acids into a transcriptional inducer converts it into a galactokinase. *Proc Natl Acad Sci U S A*, 97(7):3154–3159, Mar 2000.

[SGD] SGD. SGD Project. "Saccharomyces Genome Database". http://www.yeastgenome.org/ (accessed on September 1, 2010).

[SSZ+98] Paul T. Spellman, Gavin Sherlock, Michael Q. Zhang, Vishwanath R. Iyer, Kirk Anders, Michael B. Eisen, Patrick O. Brown, David Botstein, and Bruce Futcher. Comprehensive identification of cell cycle-regulated genes of the yeast saccharomyces cerevisiae by microarray hybridization. *Mol Biol Cell*, 9(12):3273–3297, Dec 1998.

[SSZ07] Dustin E Schones, Andrew D Smith, and Michael Q Zhang. Statistical significance of cis-regulatory modules. *BMC Bioinformatics*, 8:19, 2007.

[TCS+01] O. Troyanskaya, M. Cantor, G. Sherlock, P. Brown, T. Hastie, R. Tibshirani, D. Botstein, and R. B. Altman. Missing value estimation methods for DNA microarrays. *Bioinformatics*, 17(6):520–525, Jun 2001.

[Tre] Konstantin Tretyakov. G=MAT supplementary website. http://biit.cs.ut.ee/gmat (as of September 1, 2010).

[TRR02] David J Timson, Helen C Ross, and Richard J Reece. Gal3p and Gal1p interact with the transcriptional repressor Gal80p to form a complex of 1:1 stoichiometry. *Biochem J*, 363(Pt 3):515–520, May 2002.
